# Unique Transcriptional Profile of Sustained Ligand-Activated Preconditioning in Pre- and Post-Ischemic Myocardium

**DOI:** 10.1371/journal.pone.0072278

**Published:** 2013-08-21

**Authors:** Kevin J. Ashton, Amanda Tupicoff, Grant Williams-Pritchard, Can J. Kiessling, Louise E. See Hoe, John P. Headrick, Jason N. Peart

**Affiliations:** 1 Faculty of Health Sciences and Medicine, Bond University, Queensland, Australia; 2 Heart Foundation Research Centre, Griffith Health Institute, Griffith University, Queensland, Australia; Virginia Commonwealth University Medical Center, United States of America

## Abstract

**Background:**

Opioidergic SLP (sustained ligand-activated preconditioning) induced by 3–5 days of opioid receptor (OR) agonism induces persistent protection against ischemia-reperfusion (I-R) injury in young and aged hearts, and is mechanistically distinct from conventional preconditioning responses. We thus applied unbiased gene-array interrogation to identify molecular effects of SLP in pre- and post-ischemic myocardium.

**Methodology/Principal Findings:**

Male C57Bl/6 mice were implanted with 75 mg morphine or placebo pellets for 5 days. Resultant SLP did not modify cardiac function, and markedly reduced dysfunction and injury in perfused hearts subjected to 25 min ischemia/45 min reperfusion. Microarray analysis identified 14 up- and 86 down-regulated genes in normoxic hearts from SLP mice (≥1.3-fold change, FDR≤5%). Induced genes encoded sarcomeric/contractile proteins (*Myh7, Mybpc3,Myom2,Des*), natriuretic peptides (*Nppa,Nppb*) and stress-signaling elements (*Csda,Ptgds*). Highly repressed genes primarily encoded chemokines (*Ccl2,Ccl4,Ccl7,Ccl9,Ccl13,Ccl3l3,Cxcl3*), cytokines (*Il1b,Il6,Tnf*) and other proteins involved in inflammation/immunity (*C3,Cd74,Cd83, Cd86,Hla-dbq1,Hla-drb1,Saa1,Selp,Serpina3*), together with endoplasmic stress proteins (known: *Dnajb1,Herpud1,Socs3*; putative: *Il6, Gadd45g,Rcan1*) and transcriptional controllers (*Egr2,Egr3, Fos,Hmox1,Nfkbid*). Biological themes modified thus related to inflammation/immunity, together with cellular/cardiovascular movement and development. SLP also modified the transcriptional response to I-R (46 genes uniquely altered post-ischemia), which may influence later infarction/remodeling. This included up-regulated determinants of cellular resistance to oxidant (*Mgst3,Gstm1,Gstm2*) and other forms of stress (*Xirp1,Ankrd1,Clu*), and repression of stress-response genes (*Hspa1a,Hspd1,Hsp90aa,Hsph1,Serpinh1*) and *Txnip*.

**Conclusions:**

Protection via SLP is associated with transcriptional repression of inflammation/immunity, up-regulation of sarcomeric elements and natriuretic peptides, and modulation of cell stress, growth and development, while conventional protective molecules are unaltered.

## Introduction

Adjunctive cardioprotective therapy to limit myocardial damage and death during infarction or surgical I-R in ischemic heart disease (IHD) patients remains an important though elusive clinical goal [1]. IHD is the leading cause of death and healthcare expenditure in Australia, and is predicted to remain the leading global health problem in coming decades, emerging as a major issue in both developing and developed countries [2]. With population aging the impact of IHD will rise, with growing incidences of diabetes, obesity, dyslipidemia, and hypertension further contributing to IHD prevalence. Despite the enormity of this problem, we still have no clinically effective cardioprotective therapies to improve short- or long-term outcomes from myocardial ischemia, beyond essential (and timely) reperfusion. This reflects in part our incomplete understanding of mechanisms governing myocardial survival *vs*. death, and particularly how these are influenced by age, sex, disease and common pharmaceuticals [3], [4]. Unfortunately, widely studied experimental stimuli including pre- and post-conditioning may be impaired or negated with aging [5], relevant disease states such as diabetes [6], [7], obesity [8] and hypertension/hypertrophy [9], [10], and commonly applied drugs such as ß-blockers [11] and ACE inhibitors [9].

Contrasting conventional protective responses, SLP is highly effective in both young and aged myocardium, inducing protection that persists for up to a week post-stimulus and that is equivalent or superior to that with other preconditioning stimuli [12]–[15]. While potentially superior to other candidate responses, the molecular basis of SLP remains to be elucidated. Shifts in myocardial protein expression/localization are likely involved given that SLP requires 3–5 days of induction and generates persistent protection evident both *in vivo* and *ex vivo* [15]. Mechanistically, SLP is distinct from conventional protective and conditioning responses [14], [15], being G_i_ (pertussis toxin) insensitive and independent of well-established mediators including PI3K/Akt, NOS, mTOR, PKC, K_ATP_ channels and cRaf1 kinase (upstream of MEK/MAPK signaling), whereas G_s_-dependent PKA signals may contribute [14]. Given these unique features, we here applied un-biased transcriptome-wide interrogation to identify molecular changes associated with novel SLP.

## Results

### Cardiac Response to SLP

**Figure 1:**
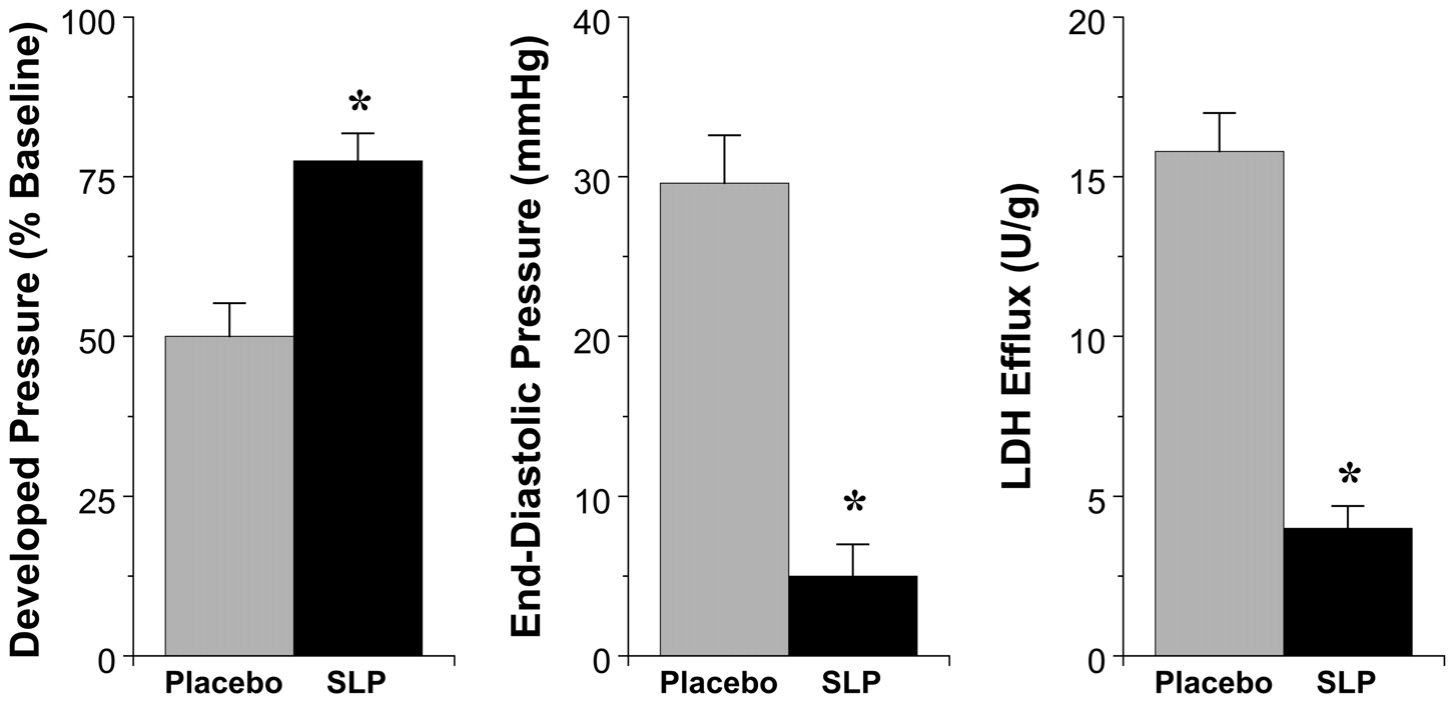
Cardioprotective effects of OR-dependent SLP. Data are shown for contractile recoveries and cell death following 25 min ischemia and 45 min reperfusion in isolated hearts from placebo *vs*. SLP treated mice (*n* = 8 per group). Shown are recoveries of left ventricular developed pressure (% of baseline) and left ventricular end-diastolic pressure (mmHg), together with total post-ischemic washout of cellular LDH. Values are mean±S.E.M. *, P<0.05 *vs.* Placebo.

Induction of SLP did not modify baseline contractile function or coronary flow in isolated perfused hearts (**Table 1**). In terms of I-R tolerance, hearts from SLP mice exhibited substantially improved ventricular contractile recoveries (**Figure 1**). Furthermore, the extent of cellular death/damage, as indicated by post-ischemic LDH efflux, was significantly reduced by >75% in the SLP group (**Figure 1**).

**Table 1:** Baseline function in Langendorff hearts from SLP and placebo mice.

GROUP	LVEDP (mmHg)	LVDP (mmHg)	+dP/dt (mmHg/s)	−dP/dt (mmHg/s)	Coronary Flow (ml/min/g)
5-day Placebo (*n* = 8)	5±1	148±9	5458±487	3345±256	3.0±0.2
5-day SLP (*n* = 8)	4±1	150±7	5220±363	3208±210	2.9±0.2

Data were acquired after 30 min aerobic perfusion (at a fixed heart rate of 420 bpm). Data are means±S.E.M. There were no significant differences in baseline (pre-ischemic) functional measures between groups. LVEDP, left ventricular end-diastolic pressure; LVDP, left ventricular developed pressure; dP/dt, differential of ventricular pressure development or relaxation over time.

### Transcriptional Effects of SLP “Induction” in Normoxic Myocardium

**Figure 2:**
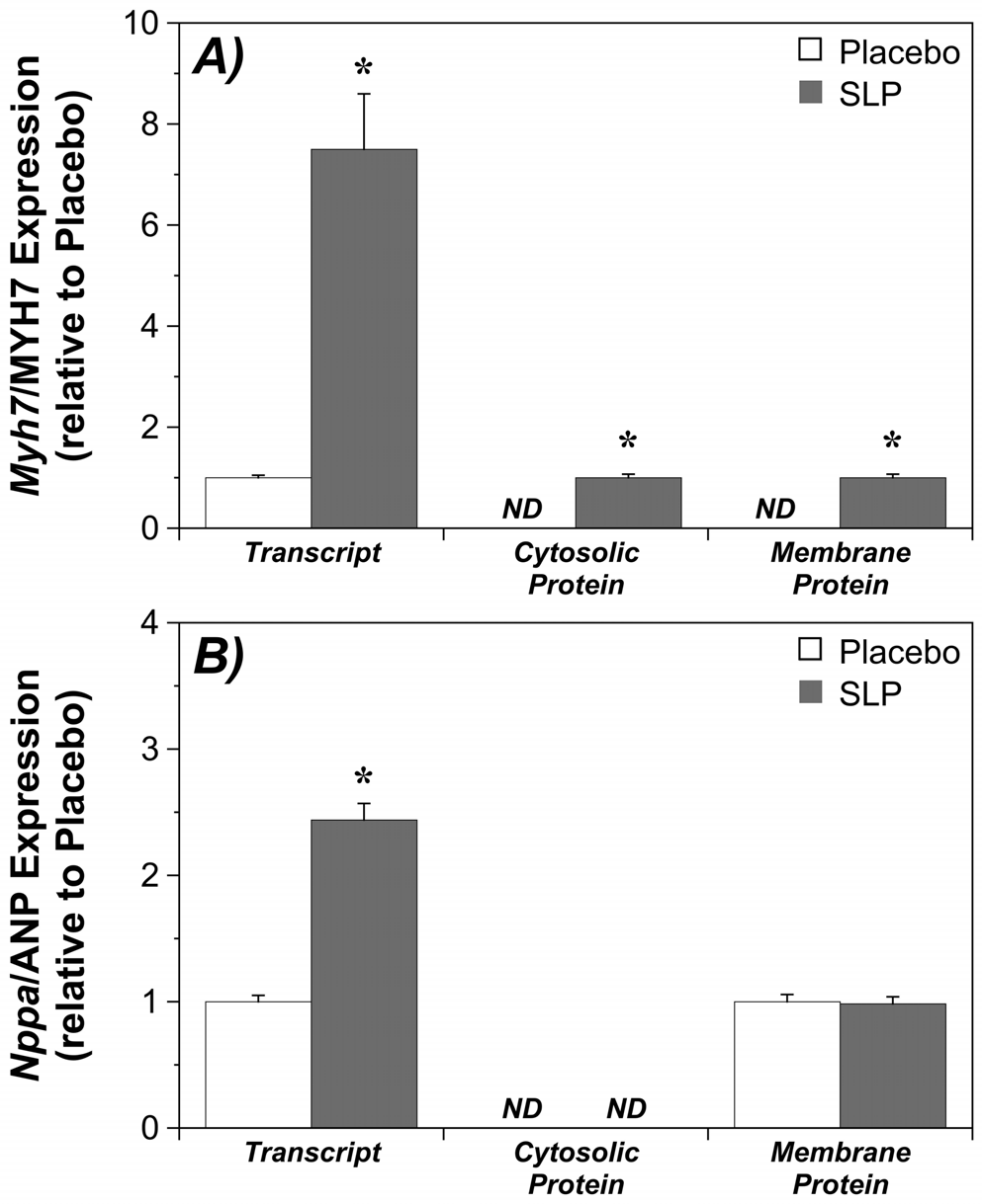
Relationship between transcript and protein expression changes for cardiac MYH7 and ANP. Data are shown for myocardial: **A)** *Myh7* and MYH7 transcript and protein levels, respectively; and **B)** *Nppa* and ANP transcript and protein levels, respectively (*n* = 6 per group). *ND*; not detected (MYH7 was un-detectable in the placebo group; ANP was un-detectable in the cytosolic fraction). Values are mean±S.E.M. *, P<0.05 *vs.* Placebo.

To identify molecular adaptations in SLP hearts, myocardial gene expression was interrogated via microarray. Of 45,200 transcripts represented on the Illumina MouseWG-6 v1.1 BeadChip, 13,335 (30%) were expressed in ≥2 myocardial samples per group. In normoxic myocardium SLP induction was associated with up-regulation of 14 transcripts and repression of 86 transcripts, based on fold-changes ≥1.3 and a FDR of ≤5% (**Table S1**). Induced transcripts were involved in contraction/sarcomeric function (*Myh7, Mybpc3, Myom2, Des*), cardioprotection/remodeling (*Nppa, Nppb*), and stress signaling (*Csda, Ptgds*). Of highly repressed transcripts, a majority were chemokines (*Ccl2, Ccl4, Ccl7, Ccl9, Ccl13, Ccl3l3, Cxcl3)*, cytokines (*Il1b, Il6, Tnf*), and other inflammation/immunity related transcripts (*Serpina3, Saa1, C3, Cd74, Hla-drb1, Hla-dbq1, Selp, Cd83, Cd86*), together with endoplasmic reticulum stress response (ERSR) genes (*Dnajb1, Socs3, Herpud1*, *Il6, Gadd45g, Rcan1*) and transcriptional controllers (*Egr2, Egr3, Fos, Hmox1, Nfkbid*). We further assessed shifts in protein expression for 2 key transcript changes - *Myh7* and *Nppa* (**Figure 2**). These data confirm *Myh7* transcript induction translates to increased myocardial MYH7 protein content (which was below detection limits in untreated tissue, consistent with normal expression in the neonatal myocardium), whereas induction of *Nppa* was not associated with a detectable increase in cardiac ANP expression (**Figure 2**).

**Figure 3:**
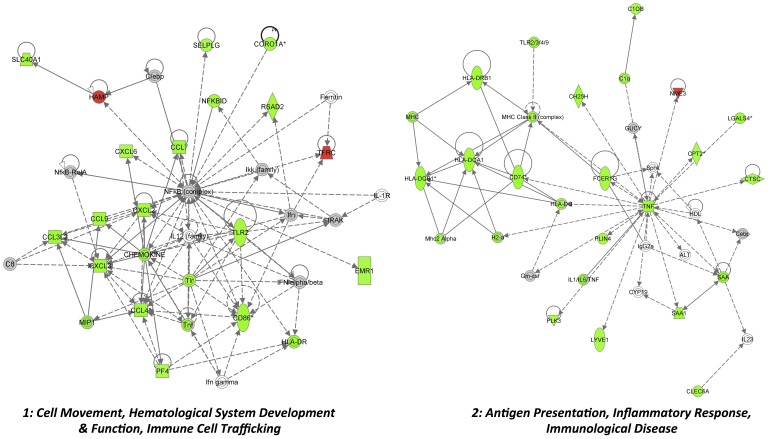
The top 2 networks modified by SLP in normoxic myocardium (networks 1 and 2, both involved in immunity/inflammation). Shown are the 2 most modified gene networks in SLP hearts. Network 1 is involved in hematological development and cellular movement/immune cell trafficking; Network 2 in antigen presentation and immune/inflammatory function. Transcripts are color-coded according to expression changes (green, up-regulated; red, down-regulated). Grey highlights molecules present in the dataset (FDR≤5%) that did not meet the ≥1.3-fold cut-off criteria. White indicates predicted molecules computationally incorporated into networks based on evidence within the IPA knowledge base. Lines between molecules indicate direct molecular connections.

**Figure 4:**
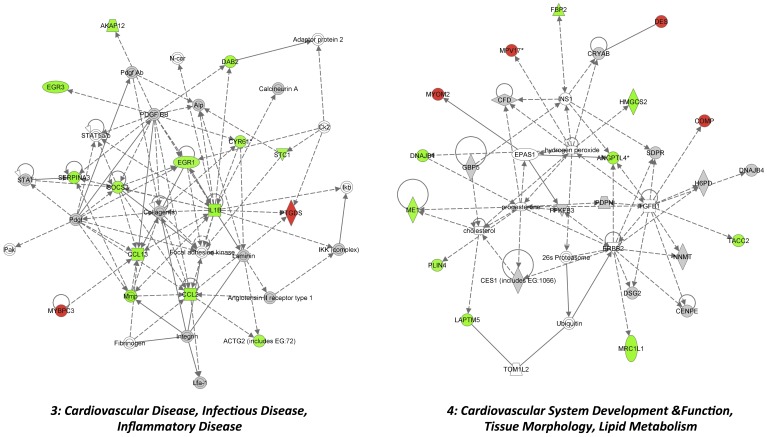
The top 2 cardiovascular-related networks modified by SLP in normoxic myocardium (networks 3 and 4). Shown are the 3rd and 4th most modified gene networks in SLP hearts. Network 3 is involved in cardiovascular, inflammatory and immune diseases; Network 4 in cardiovascular system development. Transcripts and interactions are coded as outlined in **Figure 3**.

Functional annotation and interrogation via the IPA suite identified similar themes of inflammatory/immune modulation, regulation of cell movement, growth and development, and cell death/survival responses (**Table 2** **; Table S3**). The top molecular canonical functions identified included (in descending order of significance): cell-to-cell communication and interaction, cellular movement, antigen presentation, cellular development, cellular function and maintenance, cellular growth and proliferation, cell death, and cell signaling. The top represented disease processes included: inflammatory responses, immunological disease, connective tissue disorders, inflammatory disease, skeletal and muscular disorders. These paths and functions are suggestive of SLP-dependent control of inflammatory/immune function, cardiac contraction and remodeling, and stress-responses (cell death, survival and signaling, oxidative stress responses). Network analysis identified 9 significantly modified networks during SLP induction, based upon known molecular interactions (**Table 3**). Again, the most significantly modified revolve around cell movement, immune/inflammatory functions and cardiovascular disease and development (**Table 3**). As detailed in **Figure 3** the two top modified networks are involved in inflammatory/immune function, network 1 centered on NfκB and Il12/chemokine responses, and network 2 centered on TNFα and MHC/HLA responses. The two cardiovascular-related networks identified (3 and 4) also involve inflammatory signaling, together with processes of cellular growth and development (**Figure 4**).

**Table 2:** The top functional gene groupings sensitive to SLP induction in normoxic myocardium.

Molecular and Cellular Functions	*P*-Value Range	No. of Genes
Cell-to-Cell Communication and Interaction	2.48E-23-2.25E-05	51
Cellular Movement	1.61E-20-2.25E-05	43
Antigen Presentation	9.5E-16-1.69E-05	31
Cellular Development	5.97E-14-2.62E-05	51
Cellular Function and Maintenance	6.79E-13-1.13E-05	35
Cellular Growth and Proliferation	2.82E-12-2.62E-05	59
Cell Death	1.09E-10-2.31E-05	53
Cell Signaling	7.27E-10-1.29E-05	24
Molecular Transport	7.27E-10-2.2E-05	27
**Physiological System Development and Function**	***P*** **-Value Range**	**No. of Genes**
Hematological System Development and Function	1.61E-20-2.58E-05	52
Immune Cell Trafficking	1.61E-20-2.58E-05	42
Tissue Development	2.54E-15-2.58E-05	50
Lymphoid Tissue Structure and Development	8.17E-15-1.16E-05	23
Tissue Morphology	1.25E-14-1.81E-05	35
**Disease and Disorders**	***P*** **-Value Range**	**No. of Genes**
Inflammatory Response	1.61E-20-2.58E-05	49
Immunological Disease	7.27E-17-1.84E-05	46
Connective Tissue Disorders	1.18E-13-2.47E-05	39
Inflammatory Disease	1.18E-13-2.47E-05	50
Skeletal and Muscular Disorders	1.18E-13-2.47E-05	52

Functional groupings of transcripts differentially modified by SLP in normoxic tissue (also shown are P-values, and numbers of involved genes). Groupings from IPA analysis are categorized into molecular and cellular functions, physiological system development and function, and disease and disorder (complete functional gene grouping data can be found in **Table S3**).

**Table 3:** Functional gene networks modified during SLP induction in normoxic myocardium.

*ID*	*Top Functions*	*Molecules In Network*	*Score*	*Focus Molecules*
1	Cellular Movement, Hematological System Development and Function, Immune Cell Trafficking	C8,C/ebp,CCL4,CCL7,CCL9,CCL3L3,CD86,CHEMOKINE,CORO1A,CXCL2,CXCL3,CXCL6,EMR1,Ferritin,HAMP,HLA-DR,Ifn,IFN alpha/beta,Ifn gamma,Ikk (family),IL-1R,IL12 (family),IRAK,MIP1,NFkB (complex),NfkB-RelA,NFKBID,PF4,RSAD2,SELPLG,SLC40A1,TFRC,Tlr,TLR2,Tnf	32	18
2	Antigen Presentation, Inflammatory Response, Immunological Disease	ALT,C1q,C1QB,CD74,Cebp,CH25H,CLEC6A,CPT2,CTSC,CYP19,FCER1G,Gm-csf,GUCY,H2-a,HDL,HLA-DQ,HLA-DQA1,HLA-DQB1,HLA-DRB1,IgG2a,IL23,IL1/IL6/TNF,LGALS4,LYVE1,MHC,MHC Class II (complex),Mhc2 Alpha,NME3,PLIN4,PLK3,SAA,SAA1,Sphk,TLR2/3/4/9,TNF	31	17
3	Cardiovascular Disease, Infectious Disease,Inflammatory Disease	ACTG2 (includes EG:72),Adaptor protein 2,AKAP12,Alp,Angiotensin II receptor type 1,Calcineurin A,CCL2,CCL13,Ck2,Collagen(s),CYR61,DAB2,EGR1,EGR3,Fibrinogen,Focal adhesion kinase,Ikb,IKK (complex),IL1B,Integrin,Laminin,Lfa-1,Mmp,MYBPC3,N-cor,Pak,Pdgf,Pdgf Ab,PDGF BB,PTGDS,SERPINA3,SOCS3,STAT,STAT5a/b,STC1	24	14
4	Cardiovascular System Development and Function, Tissue Morphology, Lipid Metabolism	26s Proteasome,ANGPTL4,CENPE,CES1 (includes EG:1066),CFD,cholesterol,COMP,CRYAB,DES,DNAJB1,DNAJB4,DSG2,EPAS1,ERBB2,FBP2,GBP5,H6PD,HMGCS2,hydrogen peroxide,INS1,LAPTM5,ME1,MPV17,MRC1L1,MYOM2,NNMT,PDPN,PFKFB3,PLIN4,progesterone,SDPR,TACC2,TGFB1,TOM1L2,Ubiquitin	21	13
5	Hematological System Development and Function, Hematopoiesis, Lymphoid Tissue Structure and Development	AMPK,CD83,Cdc2,Cyclooxygenase,Elastase,Fcer1,GADD45G,Growth hormone,Hsp27,Hsp70,IFI16,IFI27L2,IFITM1,IFN Beta,Ifnar,Ige,IL1,IL6,IL12 (complex),IRG,JAK,Ldh,LDL,NfkB1-RelA,Nos,NPC1,P38 MAPK,RCAN1,SELE,SELP,Sod,SYK/ZAP,TLR2/TLR4,UCP3,VCAM1	19	12
6	Drug Metabolism, Lipid Metabolism, Small Molecule Biochemistry	ABRA,BCL2A1,BCR,C3,Calcineurin protein(s),Calpain,Caspase,Caspase 3/7,CD72,CFP,CSDA,Cyclin A,DUSP1,Endothelin,Eotaxin,ERK1/2,GSTA3,HERPUD1,Iga,Igm,IL1A,Immunoglobulin,JINK1/2,MAP2K1/2,NPPA,NPPB,p70 S6k,Pi3-kinase,PLA2,Pld,Pro-inflammatory Cytokine,Raf,Rar,Sapk,Tgf beta	19	12
7	Lipid Metabolism, Small Molecule Biochemistry, Embryonic Development	ACTB,ADCY,Akt,ANGPTL4,Ap1,CD3,Creb,Cyclin E,EGR2,ERK,Erm,Estrogen Receptor,F Actin,FOS,FOSL2,FSH,hCG,HMOX1,Hsp90,Insulin,JUN/JUNB/JUND,Lh,Mapk,Mek,Nfat (family),NGF,p85 (pik3r),Pka,Pkc(s),PPP1R15A,RAB8B,Ras,Ras homolog,TCR,Vegf	10	8
8	Protein Degradation, Protein Synthesis, Organ Morphology	Actin,ADAMTS4,AOX1,CD74,CLPX,CNDP2,Collagen type I,CTRL,FAP,FCGR1C,GUK1,IFITM3,IgG,IgG1,IGg-Rheumatoid factor,IMMP2L,Interferon alpha,Jnk,KRAS,leukotriene D4,LGMN,MMP13,MMRN1,MYH7,PDK4,PEPD,peptidase,PEROXIDASE,PI3K,PREP,RNA polymerase II,SERPINB7,SPCS3,SPPL2B,Tni	8	6
9	Cell Cycle, Gene Expression, Cellular Growth and Proliferation	AKAP12,ASF1B,BAZ2A,BRD2,CBR2,CD69,CHFR,DIO1,DOT1L,ELP2,ELP3 (includes EG:55140),ERCC6,G6PD,Gcn5l,Hat,HIRA,Histone h3,Histone h4,HPSE,HRAS,ING1,JMJD6,KIR3DL1,MCM4,MGMT,NOC2L,PELP1,SH2D3C,SLAMF9,SMN2,SOX5 (includes EG:6660),THAP7,VRK1,ZBTB5,ZBTB7A	3	3

### Transcriptional Effects of SLP in Post-Ischemic Myocardium

Myocardial gene expression patterns following ischemic insult will influence progression of infarction, remodeling and ultimately failure. Post-ischemic expression patterns were significantly modified by SLP, which led to up-regulation of 29 transcripts and repression of 51 transcripts in reperfused myocardium (**Table S2**). Of these SLP-responsive transcripts, 33 were similarly altered by SLP pre-ischemia, whereas 46 were identified as specifically modified by SLP post-ischemic tissue only (21 up- and 25 down-regulated). The latter included up-regulation of transcripts involved in cardiac stress signaling and development (*Xirp1, Ankrd1, Clu*) and anti-oxidant function (*Mgst3, Gstm1, Gstm2*), and repression of *Txnip* and heat shock transcripts (*Hsph1, Hspa1a, Hspd1, Serpinh1*) (**Table S2**). Functional/pathway analysis of post-ischemic transcriptional responses to SLP identified processes and networks similar to those modified in normoxic tissue, including inflammatory/immune signaling paths and processes, cellular movement, growth, development, and signaling (**Tables 4** **and** **5** **; Table S4**).

**Table 4:** The top functional gene groupings sensitive to SLP in post-ischemic myocardium.

Molecular and Cellular Functions	*P*-Value Range	No. of Genes
Cell-To-Cell Signaling and Interaction	7.10E-13 - 2.00E-03	33
Cellular Movement	3.75E-08 - 2.52E-03	26
Antigen Presentation	5.52E-08 - 2.37E-03	18
Cell Signaling	5.72E-08 - 2.50E-03	19
Small Molecule Biochemistry	5.72E-08 - 2.52E-03	29
**Physiological System Development and Function**	***P*** **-Value Range**	**No. of Genes**
Hematological System Development and Function	2.80E-10 - 2.53E-03	31
Immune Cell Trafficking	5.80E-10 - 2.52E-03	26
Nervous System Development and Function	5.03E-08 - 5.88E-04	8
Endocrine System Development and Function	1.85E-07 - 2.52E-03	7
Tissue Development	5.43E-07 - 2.52E-03	31
**Disease and Disorders**	***P*** **-Value Range**	**No. of Genes**
Inflammatory Response	4.70E-10 - 2.52E-03	31
Inflammatory Disease	1.13E-09 - 2.52E-03	31
Renal and Urological Disease	1.51E-09 - 1.77E-03	18
Immunological Disease	3.80E-09 - 2.52E-03	19
Cardiovascular Disease	6.63E-09 - 2.53E-03	22

Functional groupings of transcripts differentially modified by SLP in post-ischemic tissue (also shown are P-values, and numbers of involved genes). Groupings from IPA analysis are categorized into molecular and cellular functions, physiological system development and function, and disease and disorders (complete functional gene grouping data can be found in **Table S4**).

**Table 5:** Functional gene networks modified by SLP in post-ischemic myocardium.

ID	Top Functions	Molecules In Network	Score	Focus Molecules
1	Lipid Metabolism, Small Molecule Biochemistry, Cellular Function and Maintenance	Alp,AMPK,CLU,Cyclooxygenase,Cytochrome c,DNAJB1,DUSP6,Fcer1,glutathione transferase,Growth hormone,GST,GSTM1,GSTM2,Hsp27,Hsp70,HSP90AA1,HSPA1A,IGFBP5, IgG1,IgG2a,IL1,IL6,JINK1/2,LDL,LGALS4,MGST3,NPC1,PLIN2,SAA,Serine Protease, SERPINA3,Sod,STAT,UCP2,UCP3	30	16
2	Antigen Presentation, Inflammatory Response, Immunological Disease	ANKRD1,CCL4,CCL7,CCL3L3,CD74,CHEMOKINE,CORO1A,Endothelin,Gm-csf,HLA-DQ,HLA-DQA1,HLA-DQB1,HLA-DR,HLA-DRB1,IFN alpha/beta,IFN Beta,Ifn gamma,Ifnar,IFNB1,IL-1R,IL12 (complex),IL12 (family),Immunoglobulin,LY6C1,MHC,MHC Class II (complex),Mhc2 Alpha,NFkB (complex),NfkB-RelA,NfkB1-RelA,Pro-inflammatory Cytokine,RSAD2,Tlr,TNFRSF12A,TXNIP	25	14
3	Hematological System Development and Function, Hematopoiesis, Organismal Development	ANGPT1,ATP5S,C22ORF28,C5ORF13,CBR2,CCDC80,dimethylglycine,EPO,EWSR1,FYCO1,HNF4A,HRAS,HSPH1,HTT,KNDC1 (includes EG:85442),KRAS,LAPTM5,MYH7, NDUFB2,NDUFB4,NME3,NRN1,PDK4,RAPH1,RASL11B,RTP3,SEC11C,SLC25A22,SLC44A2,SLFN12L,TLN1,TNF,TRIM15 (includes EG:89870),ZBTB11,ZNF318 (includes EG:24149)	25	14
4	Nucleic Acid Metabolism, Small Molecule Biochemistry, Endocrine System Development and Function	ABRA,Akt,C3,C4A,CCL2,CDH16,Collagen(s),Cyclin A,Cyclin E,EIF4EBP1,ERK,ERK1/2,Estrogen Receptor,Focal adhesion kinase,HES1,HMOX1,HSPD1,Ige,Igm,IL1B,Insulin,MAP2K1/2, NGF,NPPA,NPPB,p70 S6k,p85 (pik3r),Pdgf,PDGF BB,Pi3-kinase,PI3K,Pld,PTGDS,Ras,STAT5a/b	22	13
5	Cell-To-Cell Signaling and Interaction, Cellular Movement, Immune Cell Trafficking	ABAT,ACPP,ANKS1A,ARC,C11ORF82,CHAC1,CRK,CXCL6,CXCL10,ERBB2,GBP1 (includes EG:14468),GCHFR,H6PD,HNRNPK,hydrogen peroxide,IFI30,IFI47,IFIT5,IFITM2,IFITM3,IFNG, IL4I1,IRF6,IRGM2,LAMP1,MIRN324,MPV17,MRC1L1,MYBPC3,PRDX2,RBMX,SERPINH1,SLCO2B1,SP110,ZFX	16	10
6	Carbohydrate Metabolism, Cell Morphology, Cellular Assembly and Organization	ANGPTL4,Ap1,B2M,Ck2,CNKSR1,EHD4,Fibrinogen,FSH,GCK,hCG,Histone h3,Histone h4, HSPG2 (includes EG:3339),IgG,Ikb,Interferon alpha,Jnk,KLRA17,Lh,LYVE1,Mapk,MHC Class I (complex),Mmp,MMP13,Nfat (family),P38 MAPK,Pkc(s),Ras homolog,RGS5,RNA polymerase II,Rxr,SERPINB7,Tgf beta,Tnf,Vegf	11	8

**Figure 5:**
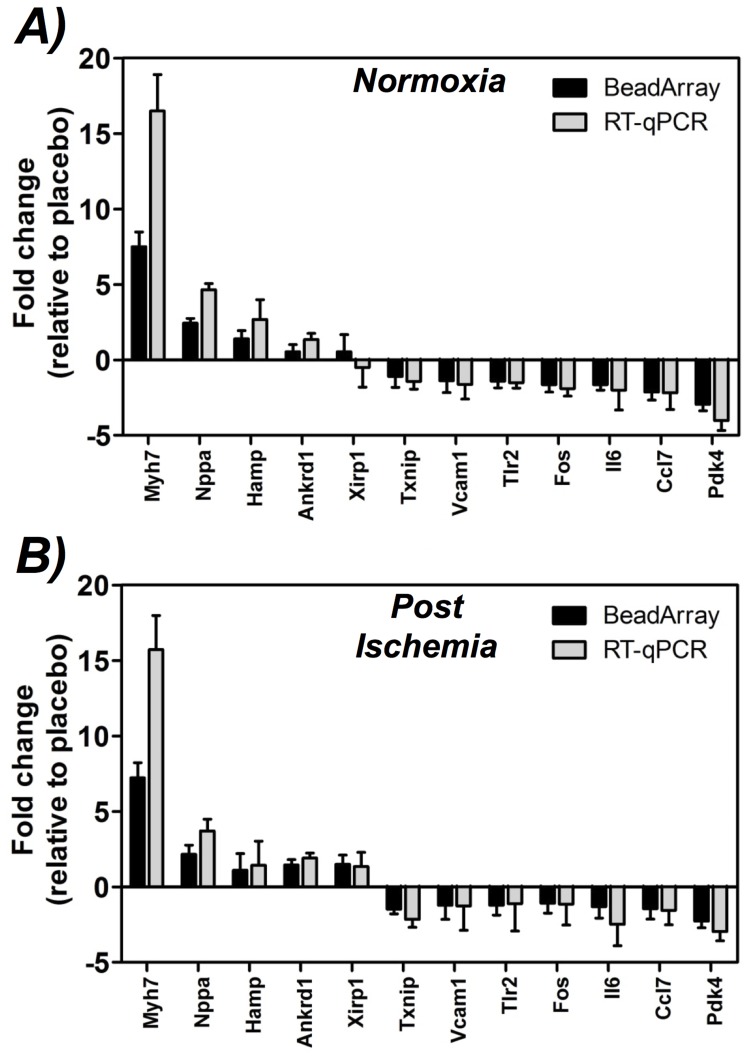
Validation of microarray assay data by RT-qPCR. Shown are expression changes determined via RT-qPCR and microarray analysis for: **A)** SLP *vs* placebo responses in normoxic myocardium; and **B)** SLP *vs.* placebo responses in post-ischemic myocardium. Data are expressed as means ± S.E.M. (*n* = 6 per group). Linear regression analysis of these data yielded a significant and strongly positive correlation (r^2^ = 0.95): RT-qPCR expression = (1.351×microarray expression) - 0.047 (the slope factor >1 indicative of a predictably greater dynamic range for RT-qPCR analysis).

Select gene changes identified via microarray interrogation of normoxic and post-ischemic myocardium were further validated via RT-qPCR analysis. As shown in **Figure 5**, genes assessed by RT-qPCR exhibited expression changes during SLP induction that were consistent with responses detected by microarray analysis. The very strong positive and linear correlation (*r* ^2^ = 0.95) highlights the complementarity of RT-qPCR and microarray techniques, and thus the general quantitative value of array-derived gene expression changes (though the slope of the relationship reflects a superior sensitivity and dynamic range for RT-qPCR; **Figure 5**). Interestingly, of this sub-set of transcriptional responses specifically quantitated by PCR, pre-ischemic induction of myocardial *Myh7* and *Nppa*, and repression of *Pdk4*, *Ccl7*, *Fos* and *Il6* have not previously been reported for cardioprotected models. Post-ischemic induction of *Ankrd1* and *Xirp1* and repression of *Txnip* and *Tlr2* have also not been linked to cardioprotection.

## Discussion

Clinically applicable interventions to limit myocardial cell death with infarction or surgical I-R are needed [3], [4]. Interventions based on conventional pre- and post-conditioning have been widely studied, yet may possess significant drawbacks. Notably, they appear sensitive to inhibitory influences of age (with the majority of IHD patients >50 yrs old), disease status (most IHD patients suffer co-morbidities of obesity/dyslipidemia, diabetes, and/or hypertension), and common pharmaceuticals (almost all IHD patients are on ß-blockers, statins, ACE inhibitors or angiotensin II receptor blockers [4]. These factors may explain modest outcomes from clinical trials of conditioning stimuli [3]. Experimental SLP on the other hand engages unique signaling which may be resistant to these inhibitory influences [14], [15], and indeed is effective in aged myocardium [13]. While SLP induction is wortmannin-sensitive [15], implicating PI3K, whether this reflects a role in phospho-dependent signal transduction, activation of mRNA translation, and/or regulation of gene transcription is not known. Certainly the resulting phenotype is unique, I-R tolerance being independent of PI3K/Akt, NOS, mTOR, K_ATP_ channel and MEK/MAPK activation [14].

Array analysis reveals SLP significantly alters the cardiac transcriptome, though consistent with novel signaling involvement [14], this does not involve modulation of canonical protective paths or molecules (*eg.* RISK signaling elements, NOS) [16], anti-oxidants, or major determinants of cell death/apoptosis. Rather, SLP induces unconventional transcriptional changes, including shifts in mediators of inflammation/immunity, sarcomere function, and cardiovascular growth and development (**Figures 3** and **4** **, Table S1**). Intriguingly, this response exhibits features similar to those arising with cardioprotective exercise [17], itself attributed to OR-dependent signaling [18], [19]. Both SLP and voluntary-running generate predominant mRNA repression *vs*. induction, and modify transcripts involved in inflammation/immunity and sarcomeric function.

### Transcripts Up-Regulated in SLP Hearts

Data in **Figure 1** highlight powerful protection against dysfunction and cell death with SLP, a persistent stress-resistance induced by several days of OR agonism [15]. This pattern implicates protein expression changes rather than or additional to post-translational regulation. Only a small set of transcripts was induced by SLP, the majority being repressed (**Table S1**). Several induced transcripts may contribute to I-R tolerance, including a novel suite of sarcomeric genes (*Myh7, Mybpc3, Myom2*, *Des*), together with potentially protective *Nppa* and *Nppb*.

#### Sarcomeric elements

*Myh7* was the most highly induced, with encoded ß-myosin heavy chain protein also elevated (**Figure 2**). There are no prior reports of OR (or ischemic) regulation of this protein, which is considered a marker of pathological hypertrophy (reflecting expression of a fetal gene program). Nonetheless, a solely pathological function for the protein has recently been challenged [20], and is contrary to improved cardiac efficiency [21] and Ca^2+^ homeostasis [22] with ß-myosin heavy chain expression. Pronounced induction may thus benefit hearts, reducing the effects of I-R on 2 key outcome determinants - contractile efficiency and Ca^2+^ handling.

Transcript for myosin-binding protein C (*Mybpc3*), a critical regulator of cardiac function, was also induced. Myosin-binding protein C stabilizes thick filaments and regulates actomyosin ATPase activity. Dysregulation leads to dilated and hypertrophic cardiomyopathies, with phospho-dependent degradation potentially contributing to I-R injury [23]. Induction has not been previously reported in protected phenotypes, yet could limit cardiac I-R injury, protect sarcomeric function, and together with ß-myosin heavy chain improve contractile efficiency.

Induced *Myom2* and *Des* may additionally preserve sarcomere function. Myomesin-2 is the primary myosin M-band cross-linking protein, and binds titin in a complex with obscurin/obs1. The protein is key to normal function, as evidenced by associations between heart failure and low expression. Since titin is a critical I-R sensitive sarcomere element [24], up-regulated myomesin-2 may mitigate against titin dysfunction (and is also increased with protective exercise [17]). Up-regulated desmin (*Des*) connects myofibrils to each other and the sarcolemma, controls mitochondrial proximity to myofibrils, and maintains myocyte structure and interactions at Z-disks/intercalated disks. Evidence indicates degradation of desmin may contribute to cardiac I-R injury [25]. Novel up-regulation may thus be protective. Collectively, induction of this suite of key sarcomeric genes may protect against I-R damage, with reduced desmin and myosin-binding protein C already implicated in I-R injury, while roles for ß-myosin heavy chain and myomesin-2 warrant further study.

#### Natriuretic peptides

Transcripts for atrial natriuretic peptide (ANP) and brain natriuretic peptide (BNP) - *Nppa* and *Nppb*, respectively - were induced with SLP. There are no prior reports of OR-dependent control of cardiac ANP/BNP expression, though the secretion of ANP may be enhanced by ORs [26]–[28]. Surprisingly, ANP/BNP involvement in pre- or post-conditioning has not been tested, despite increased secretion with brief ischemia and reduced I-R injury with exogenously applied peptide [29]–[31]. ANP and BNP appear to limit cell damage via cGMP/PKG signals, NO and K_ATP_ channels, and modulation of SR Ca^2+^ handling [31], [32]. Curiously, despite *Nppa* induction here we did not detect changes in ANP protein (**Figure 2**). The basis for these differing responses is unclear. Cardiac ANP is largely restricted to secretory granules, secretion leading to membrane-receptor and cGMP-dependent protection. It is possible SLP enhances both ANP expression and subsequent secretion, since opioidergic stimuli (including morphine and μ and κ-OR agonists) increase cardiac ANP secretion [26]–[28], which can be temporally dissociated from *Nppa* expression [26]. Potential protection via SLP-dependent ANP/BNP expression is consistent with impaired I-R tolerance in mice lacking the natriuretic peptide receptor guanylyl cyclase-A [33]. Furthermore, cardioprotection with prolonged oxytocin is associated with ANP expression [34], and post-ischemic *Nbbp* correlates with I-R tolerance in a model of epoxyeicosatrienoic acid mediated protection [35]. Nonetheless, mechanistic involvement of the peptides in these and other protective responses remains to be established.

#### Other induced transcripts

Up-regulated *Csda* encodes cold-shock domain protein A (or OxyR), a redox-sensitive transcriptional controller of anti-oxidants and cellular stress responses [36]. Induction has not been reported in protected states, yet may promote myocardial I-R tolerance. Up-regulated *Ptgds* encodes prostaglandin D2 synthase, involved in synthesis of prostaglandin D2. Induction prevents cardiovascular injury via anti-inflammatory effects [37], protects against platelet aggregation, and limits growth of vascular smooth muscle cells [38], effects that could contribute to I-R resistance *in vivo*. The function of induced *Mpv17* is poorly understood, though there is evidence this inner mitochondrial membrane protein may regulate mtDNA copy number and longevity [39]. *Tfrc* encodes the transferrin receptor, which contributes to iron handling and could facilitate iron-dependent oxidative stress. However, there is no strong link between iron handling and cell damage in myocardial infarction.

### Transcripts Repressed in SLP Hearts

The majority of SLP-sensitive transcripts were repressed (**Table S1**). Predominant transcript repression has also been observed in I-R resistant hearts from exercised animals [17]. Most repressed transcripts were involved in inflammation/immunity, supporting suppression of inflammation in I-R resistant tissue, again consistent with anti-inflammatory transcriptional patterns with exercise [17]. Additionally, a number of stress-response genes were repressed by SLP (**Table S1**).

#### Inflammation/immunity

Transcripts involved in inflammation/immunity, including interleukins, chemokines/cytokines and their receptors, and other immune modulators, were down-regulated (**Table S1**). A number of these changes may be relevant to I-R tolerance. *Ccl2* (MCP-1) was one of the most repressed, and is involved in monocyte invasion during I-R [40]. Inhibition of MCP-1 thus protects the heart, reducing monocyte infiltration and inflammation [41]. MCP-1 additionally mediates myocyte death via ER stress [42]. Repression of MCP-1 and other pro-inflammatory chemoattractants such as *Cxcl3, Ccl9*/MCP-5, *Ccl4*/MIP-1ß, and *Ccl7*/MCP-3 may thus be relevant to I-R tolerance, particularly *in vivo*. Indeed, *Ccl7* exaggerates inflammatory injury in heart [43]. The cytokine TNFα is a well-established mediator of inflammation, cell death and I-R injury, and repression of *Tnf* together with pro-inflammatory, pro-apoptotic and cardiodepressant *Il1ß* and *Il6*, may also limit inflammation and cell damage during I-R.

#### Endoplasmic Reticulum Stress-Response (ERSR) transcripts

A significant number of repressed transcripts are involved in or targeted by the ERSR. While generally beneficial, the ERSR can also promote apoptosis during severe or sustained insult, including myocardial I-R [44]. Known ERSR genes *Dnajb1, Socs3* and *Herpud1* were repressed by SLP, together with putative ERSR genes *Il6, Gadd45g* and *Rcan1*. As noted above, MCP-1 also up-regulates myocyte ER stress genes, such as *Dnajb1*, promoting ER-dependent apoptosis [42]. Repression of *Dnajb1* and MCP-1 may thus counter death signaling. Reductions in *Socs3*, a feedback inhibitor of JAK-Stat, can also limit infarction and remodeling [45]. Repression of *Il6* may further contribute since *Il6* induction by BNIP3 in hypoxia/ischemia may mediate infarction and pathological remodeling.

#### Other repressed transcripts

Other down-regulated transcripts are relevant to SLP protection. *Pdk4*, encoding pyruvate dehydrogenase kinase 4 (PDK4), was the most repressed in SLP hearts (**Table S1**). PDK4 phosphorylates and inactivates pyruvate dehydrogenase, with repression favoring a substrate switch from fatty acid to glucose metabolism, a shift known to protect against I-R injury [46], [47]. While there are no reported associations between *Pdk4* expression and cardioprotection, inhibition of PDK does protect ischemic myocardium [48], supporting benefit via SLP-dependent *Pdk4* repression. Repression of *Mmp13* may also improve post-ischemic outcomes since MMP-13 is involved in post-infarction fibrosis and detrimental ventricular remodeling.

### SLP Modulation of Post-Ischemic Transcripts

While ∼1/3 of transcripts modified by SLP in post-ischemic tissue were similarly altered prior to ischemia, 46 were specific to post-ischemic tissue (**Table S2**). These include up-regulated transcripts for regulators of cardiac growth and function (xin actin-binding repeat containing 1 and ankyrin repeat domain 1 - repression of the latter linked to cardiac apoptosis), anti-oxidants and cell-stress proteins (microsomal glutathione S-transferase 3, glutathione S-transferase µ1 and µ2, clusterin), cell-signaling elements (dual specificity phosphatase 6, connector enhancer of kinase suppressor of Ras 1, GTP cyclohydrolase I feedback regulator), metabolic enzymes (glucokinase or hexokinase-4, NADH dehydrogenase 1ß sub-complex 4), and tumor necrosis factor receptor superfamily member 12A (TWEAK receptor, involved in maladaptive remodeling and inflammatory disease). Transcripts repressed in post-ischemic tissue included *Txnip*, which inhibits thioredoxin and is an important determinant of myocardial I-R damage not previously linked to cardioprotective stimuli. *Ucp2* was also repressed, which may protect against oxidative stress. A number of these responses, together with common pre- and post-ischemic responses outlined above (*eg*. induced *Myh7, Mybpc3, Nppa, Nppb*; repressed *Pdk4, Ccl2, Ccl4, Il1ß, Ccl7*) could well contribute to improvements in post-ischemic infarct development with SLP.

SLP also repressed post-ischemic expression of a cluster of stress-responsive transcripts, consistent with enhanced I-R tolerance (**Figure 1**). Post-ischemic expression of *Hsph1, Hspa1a, Serpinh1, Hspd1,* and *Hsp90aa1* was reduced in SLP hearts (**Table S2**). As the cells molecular response to insult determines stress-signaling activation (rather than the nature of the external insult itself), reduced expression of such transcripts may well reflect a more robust intracellular milieu and intrinsic resistance to I-R in SLP hearts.

## Conclusions

Consistent with a unique signaling profile [14], SLP does not transcriptionally modify canonical pathways/mediators of cell survival and cardioprotection, but regulates expression of genes involved in inflammation/immunity, contractile/sarcomeric function, cardiac growth and development, and stress-signaling. The novel SLP response offers distinct advantages as a candidate for development of adjunctive cardioprotection (albeit currently limited to pre-ischemic intervention), including preserved efficacy in aged myocardium [13] where conventional stimuli may fail [4]. Though able to limit infarction up to 40% if implemented within a 60–90 min window from symptom onset [16], effects of reperfusion therapy remain variable: in as many as half of patients, 50% of at-risk myocardium may not be salvaged, while in a quarter up to 75% infarction may still occur [49]. Unfortunately, optimal timing of reperfusion is frequently unrealized, with reports of delays of 4–5 hrs from symptom onset to reperfusion for US patients [50], [51], and in urban Australian hospitals [52]. These data emphasize the need for adjunctive cardioprotection that may be applied prior to, with or after reperfusion to improve outcomes, broaden the window for reperfusion and limit progression to failure. The present data, highlighting shifts in inflammation/immunity and sarcomere structure/function with SLP, suggest that modulation of inflammatory signaling and sarcomeric integrity may be valuable in generating sustained I-R tolerance. Future work is warranted in identifying the functional relevance of these novel gene expression patterns in governing myocardial resistance to injurious stimuli.

## Materials and Methods

All studies were approved by and performed in accordance with the guidelines of the Animal Ethics Committee of Griffith University, which is accredited by the Queensland Government, Department of Primary Industries and Fisheries under the guidelines of “The Animal Care and Protection Act 2001, Section 757”.

### Animals and Experimental Design

Mature male C57Bl/6 mice aged 10–14 weeks (*n* = 8 for functional studies; *n* = 6 for gene and protein expression analyses) were briefly anesthetized with halothane, a small incision made at the base of the neck, and placebo or 75-mg morphine pellets (National Institute of Drug Abuse, Bethesda, MD) were inserted into the dorsal subcutaneous space before closure with 9-mm wound clips, as outlined previously [12], [15]. Pellets were left in place for 5 days before analysis of cardiac I-R tolerance *ex vivo* and analysis of cardiac gene expression.

### Ex Vivo Myocardial I-R

Mice were anesthetized with sodium pentobarbital (60 mg/kg) and hearts excised and perfused in a Langendorff mode as described previously [15]. After 30 min stabilization control (placebo) and SLP hearts were subjected to either 25 min of global normothermic ischemia and 45 min of aerobic reperfusion, or time-matched normoxic perfusion. Protection afforded by SLP was evaluated by assessing post-ischemic recoveries of left ventricular end-diastolic pressure and developed pressures, with total washout of myocardial LDH throughout the reperfusion period employed as an indicator of cellular disruption/oncosis (LDH content assayed enzymatically as outlined previously [15]). On completion of experiments hearts were stored in cold RNA*later* solution to protect RNA integrity and expression levels prior to ventricular dissection and RNA extraction.

### RNA Isolation and Microarray Analysis

Microarray analysis was performed in a manner similar to that outlined previously [17]. Atrial and vascular tissue was removed and left ventricular myocardium dissected from each heart, homogenized in TRIzol® reagent (Invitrogen, Carlsbad, CA, USA), and total RNA isolated according to manufacturer’s guidelines. Total RNA was further purified using RNeasy spin columns (Qiagen, Maryland, USA). The RNA yield and integrity were determined using a NanoDrop ND-1000 (NanoDrop Technologies, Wilmington, DE, USA) and a 2100 Bioanalyzer (Agilent Technologies, Forest Hills VIC, Australia), respectively. RNA integrity (RIN) scores were ≥8.0 in each sample.

Microarray experiments were performed at the IMB Microarray Facility (University of Queensland) according to standard protocols. In brief, 0.5 µg of total RNA was used to synthesize biotinylated amplified RNA (aRNA) using an Illumina TotalPrep RNA amplification kit (Illumina Inc., La Jolla, CA, USA). Samples of aRNA (1.5 µg) were fragmented and hybridized (*n* = 6 per group) to MouseWG-6 v1.1 BeadChips (Illumina Inc., La Jolla, CA, USA). Following hybridization, microarrays were washed and stained with streptavidin-Cy3 prior to scanning on an Illumina BeadStation Scanner. Data values with detection scores were compiled using BeadStudio v2.3.41 (Illumina Inc., La Jolla, CA, USA). The data discussed here were deposited into NCBI’s Gene Expression Omnibus (GEO). Data are accessible through GEO Series accession number GSE39407 at http://www.ncbi.nlm.nih.gov/geo/.

### Array Data Analysis

Microarray expression data were variance stabilized and robust spline normalized using the ‘lumi’ package in R/BioConductor (http://www.r-project.org/) [53]. Data were filtered to include only transcripts with detection scores ≥0.99 on ≥2 arrays before statistical analysis via TIGR MeV 4.0 software (13,335 bead types passed these criteria). The Significant Analysis of Microarrays (SAM) algorithm was used to correct for multiple comparisons and non-parametrically identify differentially expressed genes [54]. After multiclass SAM analysis, transcripts with fold-changes of ≥1.3 and a false discovery rate (FDR) of ≤5% were classed as significantly differentially expressed. These genes were functionally annotated via Ingenuity Pathway Analysis (IPA) (v8.7; Ingenuity® Systems, Redwood City, CA, USA) to link SLP-sensitive genes in signal networks based on known molecule interactions and canonical pathways. IPA was also used to identify the top biological and molecular themes on the basis of over-representation analysis. Briefly, the fraction of altered genes within a canonical path was compared to the fraction of total genes within that path. Probability of involvement of the respective number of modified transcripts in the path/network is expressed as a P-value (with values <0.05 considered significant).

### RT-qPCR Confirmation of Microarray Data

Two-step RT-qPCR, utilizing SYBR Green I, was employed to confirm differential gene expression of the following 12 transcripts: *Ankrd1, Ccl7, Fos, Hamp, Il6, Myh7, Nppa, Pdk4, Tlr2, Txnip, Vcam1* and *Xirp1* (primer details provided in **Table S5**). Six additional genes (*Actb, Top1, Pgk1, Gapdh, 18S rRNA and Atp5b*) were assessed using GeNorm to determine their usability as reference genes [55]. Following GeNorm assessment, *Pgk1* was found to be the most stable (M = 0.04) and therefore served as the endogenous reference control for all mRNAs assessed via RT-qPCR. Briefly, 1 µg total RNA was used to synthesize cDNA using the Superscript III First-Strand Synthesis System (Invitrogen, Carlsbad, CA, USA) according to the manufacturer’s protocol. RT-qPCR was performed in a CFX96 Real-Time PCR Detection System (Bio-Rad, Hercules, CA). The final reaction volume (10 µL) included 5 µL iQ SYBR-Green Supermix (Bio-Rad, Hercules, CA), 100 nM of each primer, and 4 µL of a 1∶20 dilution of cDNA. Optimal qPCR cycling conditions entailed an initial denaturation at 95°C for 3 min followed by 40 cycles of 95°C for 15 sec/62°C for 60 sec. After the final PCR cycle, reactions underwent melt curve analysis to detect non-specific amplicons. All reactions were performed in triplicate with each plate containing an equal number of samples from each group, a calibrator control derived from a pool of all cDNA samples, and a no-template control. PCR amplification efficiencies (90–110%) for each primer pair were calculated using a 5-log serial dilution of calibrator sample. PCR data were analyzed using CFX Manager v1.6 (Bio-Rad, Hercules, CA). Baseline subtractions and threshold settings above background were applied to all data. The calibrator sample was used to normalize inter-assay variations, with the threshold coefficient of variance for intra- and inter-assay replicates <1% and <5%, respectively. Normalized expression (ΔΔCq) was calculated, with mRNAs normalized to *Pgk1* levels and the calibrator control then log_2_-transformed.

### Myocardial Protein Expression

To assess the impact of transcriptional changes on protein expression immunoblot analysis was employed as outlined previously [14], [15] to assess myocardial expression of MYH7 and ANP, both transcriptionally induced with SLP and implicated in modulation of cardiac phenotype under other conditions. Briefly, a sub-set of placebo and SLP hearts (*n* = 6 per group) were removed from the chest, frozen in liquid N_2_, and homogenized using a glass dounce in lysis buffer containing protease and phosphatase inhibitors. Samples containing 30 µg of protein from either cytosolic or detergent-soluble membrane fractions were loaded onto 10% acrylamide gels (equal loading confirmed by Ponceau staining) and electrophoresed at 150 V for 1.5 hrs. Protein was transferred to polyvinylidene difluoride membranes and blocked in 5% skim milk powder in Tris-buffered saline with Tween 20 (TBST) for 60 min. Membranes were then incubated with primary antibody (MYH7 or ANP; 1∶1000 dilution, Cell Signaling Technology Inc., Danvers, MA, USA) overnight at 4°C. Following 3 washes in TBST, membranes were incubated with secondary antibody and visualized on a ChemiDoc XRS system (Bio-Rad, Hercules, CA, USA). Protein expression was normalized to values for placebo hearts for the purposes of comparison.

### Statistical Analyses

Unless stated otherwise, physiological and gene expression data are expressed as means ± SEM. Statistical approaches to microarray interrogation are detailed above. Other data were analyzed using SPSS 18.0 for Windows (SPSS Inc., Chicago, IL). Comparisons between groups were made via an analysis of variance (ANOVA), with Tukeys post-hoc test applied where differences were detected. Significance was accepted for P<0.05.

## Supporting Information

Table S1 **Genes significantly modified during SLP induction in normoxic myocardium.**
(DOCX)

Table S2 **Effects of SLP on post-ischemic gene expression.**
(DOCX)

Table S3 **Functional gene groupings sensitive to SLP induction in normoxic myocardium.**
(XLSX)

Table S4 **Functional gene groupings sensitive to SLP in post-ischemic myocardium.**
(XLSX)

Table S5 **RT-qPCR primer sequences for validated targets.**
(DOCX)

## References

[1] Kloner RA, Schwartz Longacre L (2011) State of the science of cardioprotection: Challenges and opportunities - proceedings of the 2010 NHLBI Workshop on Cardioprotection. J Cardiovasc Pharmacol Ther 16: 223–232. 21821520 10.1177/1074248411402501

[2] World Health Organisation (2011). Global atlas on cardiovascular disease prevention and control. Editors: Shanthi Mendis, Pekka Puska, Bo Norrving (WHO; World Heart Federation; World Stroke Organization).

[3] Ferdinandy P, Schulz R, Baxter GF (2007) Interaction of cardiovascular risk factors with myocardial ischemia/reperfusion injury, preconditioning, and postconditioning. Pharmacol Rev 59: 418–458. 18048761 10.1124/pr.107.06002

[4] Peart JN, Headrick JP (2009) Clinical cardioprotection and the value of conditioning responses. Am J Physiol Heart Circ Physiol 296: H1705–H1720. 19363132 10.1152/ajpheart.00162.2009

[5] Schulman D, Latchman DS, Yellon DM (2001) Effect of aging on the ability of preconditioning to protect rat hearts from ischemia-reperfusion injury. Am J Physiol Heart Circ Physiol 281: H1630–H1636. 11557553 10.1152/ajpheart.2001.281.4.H1630

[6] Przyklenk K, Maynard M, Greiner DL, Whittaker P (2011) Cardioprotection with postconditioning: loss of efficacy in murine models of type-2 and type-1 diabetes. Antioxid Redox Signal 14: 781–790. 20578962 10.1089/ars.2010.3343 PMC3052273

[7] Kristiansen SB, Løfgren B, Støttrup NB, Khatir D, Nielsen-Kudsk JE, et al (2004) Ischaemic preconditioning does not protect the heart in obese and lean animal models of type 2 diabetes. Diabetologia 47: 1716–1721. 15480537 10.1007/s00125-004-1514-4

[8] Bouhidel O, Pons S, Souktani R, Zini R, Berdeaux A, et al (2008) Myocardial ischemic postconditioning against ischemia-reperfusion is impaired in ob/ob mice. Am J Physiol Heart Circ Physiol 295: H1580–H1586. 18689499 10.1152/ajpheart.00379.2008 PMC2759460

[9] Penna C, Tullio F, Moro F, Folino A, Merlino A, et al (2010) Effects of a protocol of ischemic postconditioning and/or captopril in hearts of normotensive and hypertensive rats. Basic Res Cardiol 105: 181–192. 20012872 10.1007/s00395-009-0075-6

[10] Takeuchi T, Ishii Y, Kikuchi K, Hasebe N (2011) Ischemic preconditioning effect of prodromal angina is attenuated in acute myocardial infarction patients with hypertensive left ventricular hypertrophy. Circ J 75: 1192–1199. 21403415 10.1253/circj.cj-10-0906

[11] Suematsu Y, Anttila V, Takamoto S, del Nido P (2004) Cardioprotection afforded by ischemic preconditioning interferes with chronic betablocker treatment. Scand Cardiovasc J 38: 293–299. 15513313 10.1080/14017430410021507

[12] Peart JN, Gross GJ (2004) Morphine-tolerant mice exhibit a profound and persistent cardioprotective phenotype. Circulation 109: 1219–1222. 14993125 10.1161/01.CIR.0000121422.85989.BD

[13] Peart JN, Gross GJ (2004) Chronic exposure to morphine produces a marked cardioprotective phenotype in aged mouse hearts. Exp Gerontol 39: 1021–1026. 15236761 10.1016/j.exger.2004.03.038

[14] Peart JN, Gross GJ (2006) Cardioprotective effects of acute and chronic opioid treatment are mediated via different signaling pathways. Am J Physiol Heart Circ Physiol 291: H1746–H1753. 16731654 10.1152/ajpheart.00233.2006

[15] Peart JN, Hoe LE, Gross GJ, Headrick JP (2011) Sustained ligand-activated preconditioning via δ-opioid receptors. J Pharmacol Exp Ther 336: 274–281. 20947639 10.1124/jpet.110.172593 PMC3014309

[16] Yellon DM, Hausenloy DJ (2007) Myocardial reperfusion injury. N Engl J Med 357: 1121–1135. 17855673 10.1056/NEJMra071667

[17] Budiono BP, See Hoe LE, Peart JN, Sabapathy S, Ashton KJ, et al (2012) Voluntary running in mice beneficially modulates myocardial ischemic tolerance, signaling kinases and gene expression patterns. Am J Physiol Regul Integr Comp Physiol 302: R1091–R1100. 22378772 10.1152/ajpregu.00406.2011

[18] Dickson EW, Hogrefe CP, Ludwig PS, Ackermann LW, Stoll LL, et al (2008) Exercise enhances myocardial ischemic tolerance via an opioid receptor-dependent mechanism. Am J Physiol Heart Circ Physiol 294: H402–H408. 17951371 10.1152/ajpheart.00280.2007

[19] Galvão TF, Matos KC, Brum PC, Negrão CE, Luz PL, et al (2011) Cardioprotection conferred by exercise training is blunted by blockade of the opioid system. Clinics (Sao Paulo) 66: 151–157. 21437452 10.1590/S1807-59322011000100026 PMC3044560

[20] López JE, Myagmar BE, Swigart PM, Montgomery MD, Haynam S, et al (2011) β-myosin heavy chain is induced by pressure overload in a minor subpopulation of smaller mouse cardiac myocytes. Circ Res 109: 629–638. 21778428 10.1161/CIRCRESAHA.111.243410 PMC3166391

[21] Hoyer K, Krenz M, Robbins J, Ingwall JS (2007) Shifts in the myosin heavy chain isozymes in the mouse heart result in increased energy efficiency. J Mol Cell Cardiol 42: 214–221. 17054980 10.1016/j.yjmcc.2006.08.116 PMC4412927

[22] Rice R, Guinto P, Dowell-Martino C, He H, Hoyer K, et al (2010) Cardiac myosin heavy chain isoform exchange alters the phenotype of cTnT-related cardiomyopathies in mouse hearts. J Mol Cell Cardiol 48: 979–988. 20004663 10.1016/j.yjmcc.2009.11.018 PMC3016872

[23] Sadayappan S, Osinska H, Klevitsky R, Lorenz JN, Sargent M, et al (2006) Cardiac myosin binding protein c phosphorylation is cardioprotective. Proc Natl Acad Sci USA 103: 16918–16923. 17075052 10.1073/pnas.0607069103 PMC1636554

[24] Ali MA, Cho WJ, Hudson B, Kassiri Z, Granzier H, et al (2010) Titin is a target of matrix metalloproteinase-2: implications in myocardial ischemia/reperfusion injury. Circulation 122: 2039–2047. 21041693 10.1161/CIRCULATIONAHA.109.930222 PMC3057897

[25] Blunt BC, Creek AT, Henderson DC, Hofmann PA (2007) H_2_O_2_ activation of HSP25/27 protects desmin from calpain proteolysis in rat ventricular myocytes. Am J Physiol Heart Circ Physiol 293: H1518–H1525. 17513494 10.1152/ajpheart.00269.2006

[26] Fukui K, Iwao H, Nakamura A, Tamaki T, Abe Y (1991) Effects of water deprivation and morphine administration on atrial natriuretic peptide mRNA levels in rat auricles. Jpn J Pharmacol 57: 45–50. 1839321 10.1254/jjp.57.45

[27] Yamada K, Yoshida S, Shimada Y (1991) Atrial natriuretic polypeptide secretion via selective activation of kappa-opioid receptor: role of dynorphin. Am J Physiol 261: E293–E297. 1716056 10.1152/ajpendo.1991.261.3.E293

[28] Gutkowska J, Jankowski M, Pawlak D, Mukaddam-Daher S, Izdebski J (2004) The cardiovascular and renal effects of a highly potent mu-opioid receptor agonist, cyclo[N epsilon,N beta-carbonyl-D-Lys2,Dap5]enkephalinamide. Eur J Pharmacol 496: 167–174. 15288587 10.1016/j.ejphar.2004.06.007

[29] Inserte J, Garcia-Dorado D, Agullo L, Paniagua A, Soler-Soler J (2000) Urodilatin limits acute reperfusion injury in the isolated rat heart. Cardiovasc Res 45: 351–359. 10728355 10.1016/s0008-6363(99)00371-5

[30] Sangawa K, Nakanishi K, Ishino K, Inoue M, Kawada M, et al (2004) Atrial natriuretic peptide protects against ischemia-reperfusion injury in the isolated rat heart. Ann Thorac Surg 77: 223–227. 10.1016/s0003-4975(03)01493-0 14726067

[31] D’Souza SP, Davis M, Baxter GF (2004) Autocrine and paracrine actions of natriuretic peptides in the heart. Pharmacol Ther 101: 113–129. 14761702 10.1016/j.pharmthera.2003.11.001

[32] Burley DS, Baxter GF (2008) Evidence for SERCA and BKCa activation in BNP protection of reperfused myocardium. J Mol Cell Cardiol 44: 717.

[33] Nakanishi M, Saito Y, Kishimoto I, Harada M, Kuwahara K, et al (2005) Role of natriuretic peptide receptor guanylyl cyclase-A in myocardial infarction evaluated using genetically engineered mice. Hypertension 46: 441–447. 15998711 10.1161/01.HYP.0000173420.31354.ef

[34] Ondrejcakova M, Barancik M, Bartekova M, Ravingerova T, Jezova D (2012) Prolonged oxytocin treatment in rats affects intracellular signaling and induces myocardial protection against infarction. Gen Physiol Biophys 31: 261–270. 23047939 10.4149/gpb_2012_030

[35] Chaudhary KR, Batchu SN, Das D, Suresh MR, Falck JR, et al (2009) Role of B-type natriuretic peptide in epoxyeicosatrienoic acid-mediated improved post-ischaemic recovery of heart contractile function. Cardiovasc Res 83: 362–370. 19401302 10.1093/cvr/cvp134 PMC2701722

[36] Liu H, Colavitti R, Rovira II, Finkel T (2005) Redox-dependent transcriptional regulation. Circ Res 97: 967–974. 16284189 10.1161/01.RES.0000188210.72062.10

[37] Hirawa N, Uehara Y, Yamakado M, Toya Y, Gomi T, et al (2002) Lipocalin-type prostaglandin d synthase in essential hypertension. Hypertension 39: 449–454. 11882588 10.1161/hy0202.102835

[38] Ragolia L, Palaia T, Paric E, Maesaka JK (2003) Prostaglandin D2 synthase inhibits the exaggerated growth phenotype of spontaneously hypertensive rat vascular smooth muscle cells. J Biol Chem 278: 22175–22181. 12684506 10.1074/jbc.M302769200

[39] Viscomi C, Spinazzola A, Maggioni M, Fernandez-Vizarra E, Massa V, et al (2009) Early-onset liver mtDNA depletion and late-onset proteinuric nephropathy in Mpv17 knockout mice. Hum Mol Genet 18: 12–26. 18818194 10.1093/hmg/ddn309 PMC2644642

[40] Birdsall HH, Green DM, Trial J, Youker KA, Burns AR, et al (1997) Complement C5a, TGFbeta1, and MCP-1, in sequence, induce migration of monocytes into ischemic canine myocardium within the first one to five hours after reperfusion. Circulation 95: 684–692. 9024158 10.1161/01.cir.95.3.684

[41] Liehn EA, Piccinini AM, Koenen RR, Soehnlein O, Adage T, et al (2010) new monocyte chemotactic protein-1/chemokine CC motif ligand-2 competitor limiting neointima formation and myocardial ischemia/reperfusion injury in mice. J Am Coll Cardiol 56: 1847–1857. 21087715 10.1016/j.jacc.2010.04.066

[42] Younce CW, Kolattukudy PE (2010) MCP-1 causes cardiomyoblast death via autophagy resulting from ER stress caused by oxidative stress generated by inducing a novel zinc-finger protein, MCPIP. Biochem J 426: 43–53. 19925454 10.1042/BJ20090976

[43] Westermann D, Savvatis K, Lindner D, Zietsch C, Becher PM, et al (2011) Reduced degradation of the chemokine MCP-3 by matrix metalloproteinase-2 exacerbates myocardial inflammation in experimental viral cardiomyopathy. Circulation 124: 2082–2093. 21986287 10.1161/CIRCULATIONAHA.111.035964

[44] Toth A, Jeffers JR, Nickson P, Min JY, Morgan JP, et al (2006) Targeted deletion of Puma attenuates cardiomyocyte death and improves cardiac function during ischemia-reperfusion. Am J Physiol Heart Circ Physiol 291: H52–H60. 16399862 10.1152/ajpheart.01046.2005

[45] Oba T, Yasukawa H, Hoshijima M, Sasaki K, Futamata N, et al (2012) Cardiac-specific deletion of SOCS-3 prevents development of left ventricular remodeling after acute myocardial infarction. J Am Coll Cardiol 59: 838–852. 22361405 10.1016/j.jacc.2011.10.887

[46] Kantor PF, Dyck JR, Lopaschuk GD (1999) Fatty acid oxidation in the reperfused ischemic heart. Am J Med Sci 318: 3–14. 10408755 10.1097/00000441-199907000-00002

[47] Taegtmeyer H, Goodwin GW, Doenst T, Frazier OH (1997) Substrate metabolism as a determinant for postischemic functional recovery of the heart. Am J Cardiol 80: 3A–10A. 10.1016/s0002-9149(97)00452-9 9293950

[48] Roche TE, Hiromasa Y (2007) Pyruvate dehydrogenase kinase regulatory mechanisms and inhibition in treating diabetes, heart ischemia, and cancer. Cell Mol Life Sci 64: 830–849. 17310282 10.1007/s00018-007-6380-z PMC11136253

[49] Miura T, Miki T (2008) Limitation of myocardial infarct size in the clinical setting: current status and challenges in translating animal experiments into clinical therapy. Basic Res Cardiol 103: 501–513. 18716709 10.1007/s00395-008-0743-y

[50] Cannon CP, Gibson CM, Lambrew CT, Shoultz DA, Levy D, et al (2000) Relationship of symptom-onset-to-balloon time and door-to-balloon time with mortality in patients undergoing angioplasty for acute myocardial infarction. JAMA 283: 2941–2947. 10865271 10.1001/jama.283.22.2941

[51] Williams SC, Schmaltz SP, Morton DJ, Koss RG, Loeb JM (2005) Quality of care in U.S. hospitals as reflected by standardized measures, 2002–2004. N Engl J Med 353: 255–264. 16034011 10.1056/NEJMsa043778

[52] Dracup K, McKinley SM, Moser DK (1997) Australian patients’ delay in response to heart attack symptoms. Med J Aust 166: 233–236. 9076265 10.5694/j.1326-5377.1997.tb140101.x

[53] Du P, Kibbe WA, Lin SM (2008) lumi: a pipeline for processing Illumina microarray. Bioinformatics 24: 1547–1548. 18467348 10.1093/bioinformatics/btn224

[54] Saeed AI, Sharov V, White J, Li J, Liang W, et al (2003) TM4: a free, open-source system for microarray data management and analysis. Biotechniques 34: 374–378. 12613259 10.2144/03342mt01

[55] Everaert BR, Boulet GA, Timmermans JP, Vrints CJ (2011) Importance of suitable reference gene selection for quantitative real-time PCR: special reference to mouse myocardial infarction studies. PLoS One 6: e23793. 21858224 10.1371/journal.pone.0023793 PMC3157472

