# Supplementary material for: Unique Transcriptional Profile of Sustained Ligand-Activated Preconditioning in Pre- and Post-Ischemic Myocardium
Source: PLoS One. 2013 Aug 21;8(8):e72278. doi: 10.1371/journal.pone.0072278 (PMC3749099; doi:10.1371/journal.pone.0072278)
Supplement: Table S1 — Genes significantly modified during SLP induction in normoxic myocardium. (DOCX) [file pone.0072278.s001.docx]

**Table S1. Genes significantly modified during SLP induction in normoxic myocardium**

| **Symbol** | **Entrez Gene Name** | **Illumina ID** | **Fold Change** | **Networks** | **Location** | **Type(s)** |
| --- | --- | --- | --- | --- | --- | --- |
| **MYH7*** | myosin, heavy chain 7, cardiac muscle, beta | GI_18859640 | 7.50 | 8 | Cytoplasm | enzyme |
| PTGDS | prostaglandin D2 synthase 21kDa (brain) | GI_35215297 | 2.55 | 3 | Cytoplasm | enzyme |
| **NPPA*** | natriuretic peptide precursor A | GI_38079036 | 2.44 | 6 | Extracellular Space | other |
| MYBPC3 | myosin binding protein C, cardiac | GI_6678975 | 1.45 | 3 | Cytoplasm | other |
| TFRC | transferrin receptor (p90, CD71) | GI_34328492 | 1.41 | 1 | Plasma Membrane | transporter |
| MYOM2 | myomesin (M-protein) 2, 165kDa | GI_6678995 | 1.40 | 4 | Cytoplasm | other |
| NPPB | natriuretic peptide precursor B | GI_31982151 | 1.39 | 6 | Extracellular Space | other |
| **HAMP*** | hepcidin antimicrobial peptide | GI_14211541 | 1.39 | 1 | Extracellular Space | other |
| CSDA | cold shock domain protein A | GI_20806533 | 1.34 | 6 | Nucleus | transcription regulator |
| COMP | cartilage oligomeric matrix protein | GI_7710009 | 1.34 | 4 | Extracellular Space | other |
| MPV17 | MpV17 mitochondrial inner membrane protein | GI_6678925 | 1.33 | 4 | Cytoplasm | other |
| NME3 | non-metastatic cells 3, protein expressed in | GI_9790120 | 1.31 | 2 | Cytoplasm | kinase |
| DES | desmin | GI_33563249 | 1.31 | 4 | Cytoplasm | other |
| C2ORF40 | chromosome 2 open reading frame 40 | GI_13195693 | 1.31 |  | Extracellular Space | other |
| CPT2 | carnitine palmitoyltransferase 2 | GI_6753513 | -1.32 | 2 | Cytoplasm | enzyme |
| SELPLG | selectin P ligand | GI_31982018 | -1.32 | 1 | Plasma Membrane | other |
| CXCL6 | chemokine (C-X-C motif) ligand 6 (granulocyte chemotactic protein 2) | GI_6677886 | -1.32 | 1 | Extracellular Space | cytokine |
| EMR1 | egf-like module containing, mucin-like, hormone receptor-like 1 | GI_33859545 | -1.32 | 1 | Plasma Membrane | G-protein coupled receptor |
| PF4 | platelet factor 4 | GI_9910485 | -1.32 | 1 | Extracellular Space | cytokine |
| LYVE1 | lymphatic vessel endothelial hyaluronan receptor 1 | GI_31982380 | -1.32 | 2 | Plasma Membrane | transmembrane receptor |
| EGR1 | early growth response 1 | GI_24475900 | -1.33 | 3 | Nucleus | transcription regulator |
| IL1A | interleukin 1, alpha | GI_47087136 | -1.33 | 6 | Extracellular Space | cytokine |
| RAB8B | RAB8B, member RAS oncogene family | GI_40254271 | -1.33 | 7 | Cytoplasm | enzyme |
| NPC1 | Niemann-Pick disease, type C1 | GI_6679103 | -1.33 | 5 | Cytoplasm | transporter |
| RCAN1 | regulator of calcineurin 1 | GI_31542573 | -1.33 | 5 | Nucleus | transcription regulator |
| STC1 | stanniocalcin 1 | GI_31543781 | -1.33 | 3 | Extracellular Space | kinase |
| HERPUD1 | homocysteine-inducible, endoplasmic reticulum stress-inducible, ubiquitin-like domain member 1 | GI_11612514 | -1.33 | 6 | Cytoplasm | other |
| ACTG2(includes EG:72) | actin, gamma 2, smooth muscle, enteric | GI_31982518 | -1.35 | 3 | Cytoplasm | other |
| ACTB | actin, beta | GI_6671508 | -1.35 | 7 | Cytoplasm | other |
| CYR61 | cysteine-rich, angiogenic inducer, 61 | GI_6753593 | -1.35 | 3 | Extracellular Space | other |
| C1QB | complement component 1, q subcomponent, B chain | GI_6753219 | -1.35 | 2 | Extracellular Space | other |
| PLK3 | polo-like kinase 3 (Drosophila) | GI_23956069 | -1.35 | 2 | Nucleus | kinase |
| CLEC6A | C-type lectin domain family 6, member A | GI_9910157 | -1.37 | 2 | Plasma Membrane | other |
| CTSC | cathepsin C | GI_31560606 | -1.37 | 2 | Cytoplasm | peptidase |
| FCER1G | Fc fragment of IgE, high affinity I, receptor for; gamma polypeptide | GI_40254589 | -1.37 | 2 | Plasma Membrane | transmembrane receptor |
| IFITM1 | interferon induced transmembrane protein 1 (9-27) | GI_40254488 | -1.37 | 5 | Plasma Membrane | other |
| ME1 | malic enzyme 1, NADP(+)-dependent, cytosolic | GI_6678911 | -1.37 | 4 | Cytoplasm | enzyme |
| GADD45G | growth arrest and DNA-damage-inducible, gamma | GI_6753937 | -1.37 | 5 | Nucleus | other |
| PLIN4 | perilipin 4 | GI_10181203 | -1.37 | 2,4 | Cytoplasm | other |
| SOCS3 | suppressor of cytokine signaling 3 | GI_31982458 | -1.37 | 3 | Cytoplasm | other |
| IFI27L2 | interferon, alpha-inducible protein 27-like 2 | GI_44771123 | -1.37 | 5 | unknown | other |
| ABRA | actin-binding Rho activating protein | GI_31341947 | -1.37 | 6 | Cytoplasm | transcription regulator |
| PPP1R15A | protein phosphatase 1, regulatory (inhibitor) subunit 15A | GI_6678977 | -1.37 | 7 | Cytoplasm | other |
| ADAMTS4 | ADAM metallopeptidase with thrombospondin type 1 motif, 4 | GI_27370273 | -1.37 | 8 | Extracellular Space | peptidase |
| **VCAM1*** | vascular cell adhesion molecule 1 | GI_31981429 | -1.37 | 5 | Plasma Membrane | other |
| CORO1A | coronin, actin binding protein, 1A | GI_31982807 | -1.41 | 1 | Cytoplasm | other |
| TACC2 | transforming, acidic coiled-coil containing protein 2 | GI_45827758 | -1.41 | 4 | Nucleus | other |
| **TLR2*** | toll-like receptor 2 | GI_31981332 | -1.41 | 1 | Plasma Membrane | transmembrane receptor |
| HLA-DQA1 | major histocompatibility complex, class II, DQ alpha 1 | GI_31981715 | -1.41 | 2 | Plasma Membrane | transmembrane receptor |
| AKAP12 | A kinase (PRKA) anchor protein 12 | GI_13626039 | -1.41 | 3,9 | Cytoplasm | transporter |
| DUSP1 | dual specificity phosphatase 1 | GI_7305422 | -1.43 | 6 | Nucleus | phosphatase |
| SLAMF9 | SLAM family member 9 | GI_31982609 | -1.43 | 9 | Extracellular Space | other |
| RSAD2 | radical S-adenosyl methionine domain containing 2 | GI_31543945 | -1.43 | 1 | unknown | enzyme |
| CD72 | CD72 molecule | GI_6680889 | -1.45 | 6 | Plasma Membrane | transmembrane receptor |
| CFP | complement factor properdin | GI_38086041 | -1.45 | 6 | Extracellular Space | other |
| DAB2 | disabled homolog 2, mitogen-responsive phosphoprotein (Drosophila) | GI_12963488 | -1.45 | 3 | Plasma Membrane | other |
| FBP2 | fructose-1,6-bisphosphatase 2 | GI_6679760 | -1.45 | 4 | Cytoplasm | phosphatase |
| SLC40A1 | solute carrier family 40 (iron-regulated transporter), member 1 | GI_8394303 | -1.45 | 1 | Plasma Membrane | transporter |
| UCP3 | uncoupling protein 3 (mitochondrial, proton carrier) | GI_31543921 | -1.45 | 5 | Cytoplasm | transporter |
| GSTA3 | glutathione S-transferase alpha 3 | GI_31981723 | -1.47 | 6 | Cytoplasm | enzyme |
| SELE | selectin E | GI_6755451 | -1.47 | 5 | Plasma Membrane | other |
| AOX1 | aldehyde oxidase 1 | GI_6753067 | -1.49 | 8 | Cytoplasm | enzyme |
| CBR2 | carbonyl reductase 2 | GI_6671687 | -1.49 | 9 | Cytoplasm | enzyme |
| LAPTM5 | lysosomal protein transmembrane 5 | GI_31543110 | -1.49 | 4 | Plasma Membrane | other |
| CXCL2 | chemokine (C-X-C motif) ligand 2 | GI_6680108 | -1.49 | 1 | Extracellular Space | cytokine |
| IFI16 | interferon, gamma-inducible protein 16 | GI_31982555 | -1.52 | 5 | Nucleus | transcription regulator |
| MRC1L1 | mannose receptor, C type 1-like 1 | GI_6678931 | -1.54 | 4 | Plasma Membrane | transmembrane receptor |
| ANGPTL4 | angiopoietin-like 4 | GI_10181163 | -1.54 | 4,7 | Extracellular Space | other |
| HMOX1 | heme oxygenase (decycling) 1 | GI_6754211 | -1.54 | 7 | Cytoplasm | enzyme |
| CD83 | CD83 molecule | GI_6753355 | -1.59 | 5 | Plasma Membrane | other |
| CD86 | CD86 molecule | GI_31542363 | -1.59 | 1 | Plasma Membrane | transmembrane receptor |
| NFKBID | nuclear factor of kappa light polypeptide gene enhancer in B-cells inhibitor, delta | GI_26024318 | -1.59 | 1 | unknown | other |
| SELP | selectin P (granule membrane protein 140kDa, antigen CD62) | GI_6755455 | -1.61 | 5 | Plasma Membrane | other |
| CCL9 | chemokine (C-C motif) ligand 9 | GI_6755433 | -1.61 | 1 | Extracellular Space | cytokine |
| CXCL3 | chemokine (C-X-C motif) ligand 3 | GI_6677884 | -1.61 | 1 | Extracellular Space | cytokine |
| **FOS*** | FBJ murine osteosarcoma viral oncogene homolog | GI_31560587 | -1.64 | 7 | Nucleus | transcription regulator |
| FOSL2 | FOS-like antigen 2 | GI_40789304 | -1.64 | 7 | Nucleus | transcription regulator |
| HLA-DQB1 | major histocompatibility complex, class II, DQ beta 1 | GI_46358077 | -1.64 | 2 | Plasma Membrane | transmembrane receptor |
| HLA-DRB1 | major histocompatibility complex, class II, DR beta 1 | GI_6912403 | -1.64 | 2 | Plasma Membrane | transmembrane receptor |
| **IL6*** | interleukin 6 (interferon, beta 2) | GI_13624310 | -1.64 | 5 | Extracellular Space | cytokine |
| DNAJB1 | DnaJ (Hsp40) homolog, subfamily B, member 1 | GI_9055241 | -1.64 | 4 | Nucleus | other |
| CH25H | cholesterol 25-hydroxylase | GI_6857768 | -1.72 | 2 | Cytoplasm | enzyme |
| LGALS4 | lectin, galactoside-binding, soluble, 4 | GI_46849704 | -1.72 | 2 | Extracellular Space | other |
| CD74 | CD74 molecule, major histocompatibility complex, class II invariant chain | GI_40254578 | -1.75 | 2,8 | Plasma Membrane | transmembrane receptor |
| EGR2 | early growth response 2 | GI_23956051 | -1.75 | 7 | Nucleus | transcription regulator |
| TNF | tumor necrosis factor | GI_7305584 | -1.75 | 2 | Extracellular Space | cytokine |
| SAA1 | serum amyloid A1 | GI_42476315 | -1.75 | 2 | Extracellular Space | transporter |
| CCL13 | chemokine (C-C motif) ligand 13 | GI_6755429 | -1.75 | 3 | Extracellular Space | cytokine |
| BCL2A1 | BCL2-related protein A1 | GI_6671629 | -1.79 | 6 | Cytoplasm | other |
| EGR3 | early growth response 3 | GI_9055211 | -1.82 | 3 | Nucleus | transcription regulator |
| IL1B | interleukin 1, beta | GI_31560681 | -1.85 | 3 | Extracellular Space | cytokine |
| MMP13 | matrix metallopeptidase 13 (collagenase 3) | GI_6678895 | -1.92 | 8 | Extracellular Space | peptidase |
| CCL2 | chemokine (C-C motif) ligand 2 | GI_6755419 | -2.00 | 3 | Extracellular Space | cytokine |
| HMGCS2 | 3-hydroxy-3-methylglutaryl-CoA synthase 2 (mitochondrial) | GI_31560688 | -2.00 | 4 | Cytoplasm | enzyme |
| C3 | complement component 3 | GI_23956043 | -2.04 | 6 | Extracellular Space | peptidase |
| SERPINA3 | serpin peptidase inhibitor, clade A (alpha-1 antiproteinase, antitrypsin), member 3 | GI_6678092 | -2.08 | 3 | Extracellular Space | other |
| **CCL7*** | chemokine (C-C motif) ligand 7 | GI_42476054 | -2.13 | 1 | Extracellular Space | cytokine |
| CCL3L3 | chemokine (C-C motif) ligand 3-like 3 | GI_6755431 | -2.63 | 1 | Extracellular Space | cytokine |
| CCL4 | chemokine (C-C motif) ligand 4 | GI_7305458 | -2.78 | 1 | Extracellular Space | cytokine |
| **PDK4*** | pyruvate dehydrogenase kinase, isozyme 4 | GI_7305374 | -2.94 | 8 | Cytoplasm | kinase |

*, Differential expression verified by RT-qPCR
