# Supplementary material for: Unique Transcriptional Profile of Sustained Ligand-Activated Preconditioning in Pre- and Post-Ischemic Myocardium
Source: PLoS One. 2013 Aug 21;8(8):e72278. doi: 10.1371/journal.pone.0072278 (PMC3749099; doi:10.1371/journal.pone.0072278)
Supplement: Table S2 — Effects of SLP on post-ischemic gene expression. (DOCX) [file pone.0072278.s002.docx]

**Table S2. Effects of SLP on post-ischemic gene expression**

| **Symbol** | **Entrez Gene Name** | **Illumina ID** | **Fold Change** | **Network** | **Location** | **Type(s)** |
| --- | --- | --- | --- | --- | --- | --- |
| **CHANGED BY SLP BOTH PRE- AND POST-ISCHEMIA** | | | | | | |
| **MYH7*** | myosin, heavy chain 7, cardiac muscle, beta | GI_18859640 | 7.22 | 3 | Cytoplasm | enzyme |
| PTGDS | prostaglandin D2 synthase 21kDa (brain) | GI_35215297 | 2.34 | 4 | Cytoplasm | enzyme |
| **NPPA*** | natriuretic peptide precursor A | GI_38079036 | 2.16 | 4 | Extracellular Space | other |
| NPPB | natriuretic peptide precursor B | GI_31982151 | 1.96 | 4 | Extracellular Space | other |
| NME3 | non-metastatic cells 3, protein expressed in | GI_9790120 | 1.33 | 3 | Cytoplasm | kinase |
| MYBPC3 | myosin binding protein C, cardiac | GI_6678975 | 1.31 | 5 | Cytoplasm | other |
| MPV17 | MpV17 mitochondrial inner membrane protein | GI_6678925 | 1.30 | 5 | Cytoplasm | other |
| CBR2 | carbonyl reductase 2 | GI_6671687 | -1.32 | 3 | Cytoplasm | enzyme |
| **IL6*** | interleukin 6 (interferon, beta 2) | GI_13624310 | -1.32 | 1 | Extracellular Space | cytokine |
| HLA-DQA1 | major histocompatibility complex, class II, DQ alpha 1 | GI_31981715 | -1.33 | 2 | Plasma Membrane | transmembrane receptor |
| MRC1L1 | mannose receptor, C type 1-like 1 | GI_6678931 | -1.33 | 5 | Plasma Membrane | transmembrane receptor |
| LYVE1 | lymphatic vessel endothelial hyaluronan receptor 1 | GI_31982380 | -1.33 | 6 | Plasma Membrane | transmembrane receptor |
| NPC1 | Niemann-Pick disease, type C1 | GI_6679103 | -1.37 | 1 | Cytoplasm | transporter |
| RSAD2 | radical S-adenosyl methionine domain containing 2 | GI_31543945 | -1.37 | 2 | unknown | enzyme |
| DNAJB1 | DnaJ (Hsp40) homolog, subfamily B, member 1 | GI_9055241 | -1.41 | 1 | Nucleus | other |
| HMOX1 | heme oxygenase (decycling) 1 | GI_6754211 | -1.43 | 4 | Cytoplasm | enzyme |
| **CCL7*** | chemokine (C-C motif) ligand 7 | GI_42476054 | -1.44 | 2 | Extracellular Space | cytokine |
| HLA-DRB1 | major histocompatibility complex, class II, DR beta 1 | GI_6912403 | -1.47 | 2 | Plasma Membrane | transmembrane receptor |
| LAPTM5 | lysosomal protein transmembrane 5 | GI_31543110 | -1.47 | 3 | Plasma Membrane | other |
| UCP3 | uncoupling protein 3 (mitochondrial, proton carrier) | GI_31543921 | -1.52 | 1 | Cytoplasm | transporter |
| CORO1A | coronin, actin binding protein, 1A | GI_31982807 | -1.54 | 2 | Cytoplasm | other |
| CCL3L3 | chemokine (C-C motif) ligand 3-like 3 | GI_6755431 | -1.54 | 2 | Extracellular Space | cytokine |
| IL1B | interleukin 1, beta | GI_31560681 | -1.64 | 4 | Extracellular Space | cytokine |
| HLA-DQB1 | major histocompatibility complex, class II, DQ beta 1 | GI_46358077 | -1.67 | 2 | Plasma Membrane | transmembrane receptor |
| MMP13 | matrix metallopeptidase 13 (collagenase 3) | GI_6678895 | -1.67 | 6 | Extracellular Space | peptidase |
| SERPINA3 | serpin peptidase inhibitor, clade A (alpha-1 antiproteinase, antitrypsin), member 3 | GI_6678092 | -1.67 | 1 | Extracellular Space | other |
| C3 | complement component 3 | GI_23956043 | -1.73 | 4 | Extracellular Space | peptidase |
| CD74 | CD74 molecule, major histocompatibility complex, class II invariant chain | GI_40254578 | -1.76 | 2 | Plasma Membrane | transmembrane receptor |
| LGALS4 | lectin, galactoside-binding, soluble, 4 | GI_46849704 | -1.76 | 1 | Extracellular Space | other |
| CCL4 | chemokine (C-C motif) ligand 4 | GI_7305458 | -1.79 | 2 | Extracellular Space | cytokine |
| CCL2 | chemokine (C-C motif) ligand 2 | GI_6755419 | -2.13 | 4 | Extracellular Space | cytokine |
| **PDK4*** | pyruvate dehydrogenase kinase, isozyme 4 | GI_7305374 | -2.27 | 3 | Cytoplasm | kinase |
| ANGPTL4 | angiopoietin-like 4 | GI_10181163 | -2.44 | 6 | Extracellular Space | other |
| **CHANGED BY SLP POST-ISCHEMIA ONLY** | | | | | | |
| **XIRP1*** | xin actin-binding repeat containing 1 | GI_6756014 | 1.49 |  | Plasma Membrane | other |
| **ANKRD1*** | ankyrin repeat domain 1 (cardiac muscle) | GI_31560505 | 1.45 | 2 | Cytoplasm | transcription regulator |
| FRMD5 | FERM domain containing 5 | GI_27369971 | 1.41 | 9 | unknown | other |
| GCK | glucokinase (hexokinase 4) | GI_31982797 | 1.41 | 6 | Cytoplasm | kinase |
| DUSP6 | dual specificity phosphatase 6 | GI_13399313 | 1.38 | 1 | Cytoplasm | phosphatase |
| RASL11B | RAS-like, family 11, member B | GI_51711005 | 1.38 | 3 | unknown | enzyme |
| ABAT | 4-aminobutyrate aminotransferase | GI_37202120 | 1.36 | 5 | Cytoplasm | enzyme |
| CLU | clusterin | GI_7304966 | 1.35 | 1 | Extracellular Space | other |
| EHD4 | EH-domain containing 4 | GI_31981591 | 1.34 | 6 | Plasma Membrane | enzyme |
| TNFRSF12A | tumor necrosis factor receptor superfamily, member 12A | GI_7305058 | 1.34 | 2 | Plasma Membrane | other |
| MGST3 | microsomal glutathione S-transferase 3 | GI_13385009 | 1.33 | 1 | Cytoplasm | enzyme |
| EIF4EBP1 | eukaryotic translation initiation factor 4E binding protein 1 | GI_31542599 | 1.33 | 4 | Cytoplasm | translation regulator |
| CDH16 | cadherin 16, KSP-cadherin | GI_6680899 | 1.32 | 4 | Plasma Membrane | enzyme |
| GSTM1 | glutathione S-transferase mu 1 | GI_31982315 | 1.32 | 1 | Cytoplasm | enzyme |
| CNKSR1 | connector enhancer of kinase suppressor of Ras 1 | GI_38078880 | 1.32 | 6 | Cytoplasm | other |
| NDUFB4 | NADH dehydrogenase (ubiquinone) 1 beta subcomplex, 4, 15kDa | GI_21314825 | 1.32 | 3 | Cytoplasm | enzyme |
| SYNPO2L | synaptopodin 2-like | GI_31341052 | 1.32 |  | Cytoplasm | other |
| GSTM2 | glutathione S-transferase mu 2 (muscle) | GI_38077382 | 1.31 | 1 | Cytoplasm | enzyme |
| GCHFR | GTP cyclohydrolase I feedback regulator | GI_31342937 | 1.30 | 5 | Cytoplasm | other |
| RBMX | RNA binding motif protein, X-linked | GI_31981518 | 1.30 | 5 | Nucleus | other |
| NRN1 | neuritin 1 | GI_23956285 | 1.30 | 3 | Cytoplasm | other |
| DUSP18 | dual specificity phosphatase 18 | GI_51093846 | -1.32 | 8 | Cytoplasm | phosphatase |
| SLCO2B1 | solute carrier organic anion transporter family, member 2B1 | GI_31341583 | -1.32 | 5 | Plasma Membrane | transporter |
| SLC44A2 | solute carrier family 44, member 2 | GI_22779894 | -1.32 | 3 | Extracellular Space | transporter |
| HSPD1 | heat shock 60kDa protein 1 (chaperonin) | GI_31981678 | -1.32 | 4 | Cytoplasm | enzyme |
| PLIN2 | perilipin 2 | GI_31982515 | -1.33 | 1 | Plasma Membrane | other |
| LY6C1 | lymphocyte antigen 6 complex, locus C1 | GI_6754581 | -1.33 | 2 | Plasma Membrane | other |
| RAPH1 | Ras association (RalGDS/AF-6) and pleckstrin homology domains 1 | GI_51705041 | -1.33 | 3 | Plasma Membrane | other |
| ARC | activity-regulated cytoskeleton-associated protein | GI_9055165 | -1.33 | 5 | Cytoplasm | other |
| FYCO1 | FYVE and coiled-coil domain containing 1 | GI_22779867 | -1.35 | 3 | unknown | other |
| SLC25A22 | solute carrier family 25 (mitochondrial carrier: glutamate), member 22 | GI_21311844 | -1.35 | 3 | Cytoplasm | transporter |
| B2M | beta-2-microglobulin | GI_31981889 | -1.35 | 6 | Plasma Membrane | transmembrane receptor |
| SERPINH1 | serpin peptidase inhibitor, clade H (heat shock protein 47), member 1, (collagen binding protein 1) | GI_6753303 | -1.35 | 5 | Extracellular Space | other |
| HES1 | hairy and enhancer of split 1, (Drosophila) | GI_31560817 | -1.37 | 4 | Nucleus | transcription regulator |
| IRGM2 | immunity-related GTPase family M member 2 | GI_31980893 | -1.37 | 5 | Cytoplasm | enzyme |
| ZFAND2A | zinc finger, AN1-type domain 2A | GI_42475963 | -1.37 | 7 | unknown | other |
| IGFBP5 | insulin-like growth factor binding protein 5 | GI_6754311 | -1.39 | 1 | Extracellular Space | other |
| C5ORF13 | chromosome 5 open reading frame 13 | GI_33186876 | -1.39 | 3 | Cytoplasm | other |
| UCP2 | uncoupling protein 2 (mitochondrial, proton carrier) | GI_31543919 | -1.41 | 1 | Cytoplasm | transporter |
| C4A | complement component 4A (Rodgers blood group) | GI_27923908 | -1.41 | 4 | Extracellular Space | other |
| HSPG2 (includes EG:3339) | heparan sulfate proteoglycan 2 | GI_33636773 | -1.47 | 6 | Plasma Membrane | other |
| **TXNIP*** | thioredoxin interacting protein | GI_13994377 | -1.47 | 2 | Cytoplasm | other |
| HSP90AA1 | heat shock protein 90kDa alpha (cytosolic), class A member 1 | GI_42476088 | -1.59 | 1 | Cytoplasm | other |
| IFNB1 | interferon, beta 1, fibroblast | GI_6754303 | -1.61 | 2 | Extracellular Space | cytokine |
| HSPH1 | heat shock 105kDa/110kDa protein 1 |  | -1.70 | 3 | Cytoplasm | other |
| HSPA1A | heat shock 70kDa protein 1A | GI_50080208 | -1.70 | 1 | Cytoplasm | other |
| **DIFFERENTIALLY MODIFIED BY SLP POST- *vs.* PRE-ISCHEMIA** | | | | | | |
| ABRA | actin-binding Rho activating protein | GI_31341947 | 1.45 | 4 | Cytoplasm | transcription regulator |

*, Differential expression verified by RT-qPCR
