# Supplementary material for: Unique Transcriptional Profile of Sustained Ligand-Activated Preconditioning in Pre- and Post-Ischemic Myocardium
Source: PLoS One. 2013 Aug 21;8(8):e72278. doi: 10.1371/journal.pone.0072278 (PMC3749099; doi:10.1371/journal.pone.0072278)
Supplement: Table S5 — RT-qPCR primer sequences for validated targets. (DOCX) [file pone.0072278.s005.docx]

| Table S5. RT-qPCR primer sequences for validated targets | | | | | |
| --- | --- | --- | --- | --- | --- |
| **Gene name** | **Gene symbol** | **GenBank Accession No.** | **Forward primer (5'-3')** | **Reverse primer (5'-3')** |  |
| ankyrin repeat domain 1 (cardiac muscle) | *Ankrd1* | [NM_013468.3](http://www.ncbi.nlm.nih.gov/nuccore/NM_013468.3) | AGGACAGAGAAGGAGACAC | CCAGCACAGTTCTTGACC |  |
| chemokine (C-C motif) ligand 7 | *Ccl7* | [NM_013654.3](http://www.ncbi.nlm.nih.gov/nuccore/NM_013654.3) | GGGAAGCTGTTATCTTCAAGAC | ATGCTATAGCCTCCTCGAC |  |
| FBJ osteosarcoma oncogene | *Fos* | [NM_010234.2](http://www.ncbi.nlm.nih.gov/nuccore/NM_010234.2) | AAGCGGAGACAGATCAACTTGAAG | TCTCTTTCAGCAGATTGGCAATC |  |
| hepcidin antimicrobial peptide | *Hamp* | [NM_032541.1](http://www.ncbi.nlm.nih.gov/nuccore/NM_032541.1) | CAACAGATGAGACAGACTACAG | CAGCAGAAGATGCAGATGG |  |
| interleukin 6 | *Il6* | [NM_031168.1](http://www.ncbi.nlm.nih.gov/nuccore/NM_031168.1) | AAGCCAGAGTCCTTCAGAG | TTATCTGTTAGGAGAGCATTGG |  |
| myosin, heavy polypeptide 7, cardiac muscle, beta | *Myh7* | [NM_080728.2](http://www.ncbi.nlm.nih.gov/nuccore/NM_080728.2) | AGTCTGCTGAAGGACACTC | CACGATGGCGATGTTCTC |  |
| natriuretic peptide type A | *Nppa* | [NM_008725.2](http://www.ncbi.nlm.nih.gov/nuccore/NM_008725.2) | GCCCTCGGAGCCTACGAA | GGTACCGGAAGCTGTTGCA |  |
| pyruvate dehydrogenase kinase, isoenzyme 4 | *Pdk4* | [NM_013743.2](http://www.ncbi.nlm.nih.gov/nuccore/NM_013743.2) | CAGTAGTCCAAGATGCCTTTGAGTGT | GTTGGCCTGGAAATTTTCCATT |  |
| phosphoglycerate kinase 1 | *Pgk1* | [NM_008828.2](http://www.ncbi.nlm.nih.gov/nuccore/NM_008828.2) | AACAACATGGAGATTGGCACATC | AGGCAAGGTAATCTTCACACCATTT |  |
| toll-like receptor 2 | *Tlr2* | [NM_011905.3](http://www.ncbi.nlm.nih.gov/nuccore/NM_011905.3) | GATAATGAACACCAAGACCTACC | GCAGTTCTCAGATTTACCCA |  |
| thioredoxin interacting protein | *Txnip* | [NM_001009935.2](http://www.ncbi.nlm.nih.gov/nuccore/NM_001009935.2) | CTGTGAAGGTGATGACATCTCCAT | GCCATTGGCAAGGTAAGTGTGT |  |
| vascular cell adhesion molecule 1 | *Vcam1* | [NM_011693.3](http://www.ncbi.nlm.nih.gov/nuccore/NM_011693.3) | ACTACAAGTCTACATCTCTCCCAGGAAT | CACAGCACCACCCTCTTGAA |  |
| xin actin-binding repeat containing 1 (cardiomyopathy-associated protein 1) | *Xirp1* (*Cmya1*) | [NM_011724.3](http://www.ncbi.nlm.nih.gov/nuccore/NM_011724.3) | GTTACTCAAGGCAGCATCAG | TGGTCAGGATCTTCTGTCAC |  |
